# Enhancing Cardioprotection Through Neutrophil‐Mediated Delivery of 18β‐Glycyrrhetinic Acid in Myocardial Ischemia/Reperfusion Injury

**DOI:** 10.1002/advs.202406124

**Published:** 2024-09-12

**Authors:** Dongjian Han, Fuhang Wang, Qingjiao Jiang, Zhentao Qiao, Yuansong Zhuang, Quanxu An, Yuhang Li, Yazhe Tang, Chenyao Li, Deliang Shen

**Affiliations:** ^1^ Department of Cardiology The First Affiliated Hospital of Zhengzhou University Zhengzhou 450052 China; ^2^ Key Laboratory of Cardiac Injury and Repair of Henan Province Zhengzhou 450018 China; ^3^ Department of Vascular and Endovascular Surgery The First Affiliated Hospital of Zhengzhou University Zhengzhou 450052 China

**Keywords:** drug delivery, inflammation, myocardial ischemia‐reperfusion injury, nanomedicine, neutrophil decoys (NDs), reactive oxygen species (ROS)

## Abstract

Myocardial ischemia/reperfusion injury (MI/RI) generates reactive oxygen species (ROS) and initiates inflammatory responses. Traditional therapies targeting specific cytokines or ROS often prove inadequate. An innovative drug delivery system (DDS) is developed using neutrophil decoys (NDs) that encapsulate 18β‐glycyrrhetinic acid (GA) within a hydrolyzable oxalate polymer (HOP) and neutrophil membrane vesicles (NMVs). These NDs are responsive to hydrogen peroxide (H_2_O_2_), enabling controlled GA release. Additionally, NDs adsorb inflammatory factors, thereby reducing inflammation. They exhibit enhanced adhesion to inflamed endothelial cells (ECs) and improved penetration. Once internalized by cardiomyocytes through clathrin‐mediated endocytosis, NDs protect against ROS‐induced damage and inhibit HMGB1 translocation. In vivo studies show that NDs preferentially accumulate in injured myocardium, reducing infarct size, mitigating adverse remodeling, and enhancing cardiac function, all while maintaining favorable biosafety profiles. This neutrophil‐based system offers a promising targeted therapy for MI/RI by addressing both inflammation and ROS, holding potential for future clinical applications.

## Graphical abstract

Innovative neutrophil‐based nanodecoys deliver 18β‐glycyrrhetinic acid directly to damaged heart tissue, simultaneously tackling oxidative stress and inflammation in myocardial ischemia/reperfusion injury (MI/RI). This cutting‐edge approach not only reduces infarct size and enhances cardiac function, but also redefines the landscape of cardioprotection, offering a glimpse into the future of targeted heart therapies.

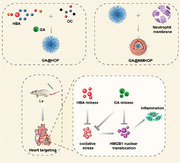

## 1 Introduction

Acute myocardial infarction (AMI), a prevalent form of coronary heart disease, has attracted significant attention due to its high prevalence and mortality rates.^[^ ^1^, ^2^, ^3^ ^]^ The most effective treatment for AMI involves immediate revascularization of the occluded artery, which is achieved through either coronary artery bypass grafting (CABG) or primary percutaneous coronary intervention (PCI).^[^ ^4^, ^5^ ^]^ However, the reperfusion process can paradoxically lead to substantial myocardial injury, primarily due to an overload of reactive oxygen species (ROS), rapid pH correction, and increase in intracellular Ca^2+^ levels.^[^ ^6^, ^7^ ^]^ This persistent redox imbalance can exacerbate cardiac inflammation, leading to further ROS production and perpetuating a deleterious cycle that worsens myocardial dysfunction.^[^ ^8^, ^9^, ^10^ ^]^ Thus, antioxidant and anti‐inflammatory therapies are potential intervention strategies for alleviating myocardial ischemia/reperfusion injury (MI/RI). However, the clinical effectiveness of traditional drugs such as antioxidants,^[^ ^11^ ^]^ anti‐inflammatory drugs,^[^ ^12^ ^]^ cardioprotective agents,^[^ ^13^ ^]^ and stem cells,^[^ ^14^ ^]^ is hampered by poor drug bioavailability and potential for systemic side effects.

18β‐Glycyrrhetinic acid (18β‐GA) is a bioactive compound derived from *Glycyrrhiza glabra*, known for its antioxidant and anti‐inflammatory properties in various diseases.^[^ ^15^, ^16^, ^17^ ^]^ It has been shown to mitigate pyrrolizidine alkaloid‐induced liver injury by enhancing the nuclear factor erythroid 2‐related factor 2 (Nrf2)‐mediated oxidative regulatory pathway.^[^ ^18^ ^]^ Additionally, 18β‐GA exhibits neuroprotective effects in ischemic stroke by reducing inflammation through the inhibition of the nuclear translocation of high‐mobility group box‐1 (HMGB1).^[^ ^19^ ^]^ However, the precise mechanisms of 18β‐GA's role in MI/RI are not entirely understood. Its clinical application is further complicated by its limited water solubility, nonspecific cardiac localization, and short biological half‐life.^[^ ^20^, ^21^, ^22^ ^]^ Moreover, long‐term intake of high doses of 18β‐GA may lead to adverse effects such as hyponatremia, hyperkalemia, hypertension, and hyperglycemia.^[^ ^23^ ^]^ Therefore, developing a precise and controllable drug delivery system (DDS) is crucial to address the therapeutic needs in cases of cardiac injury.

**Scheme 1:**
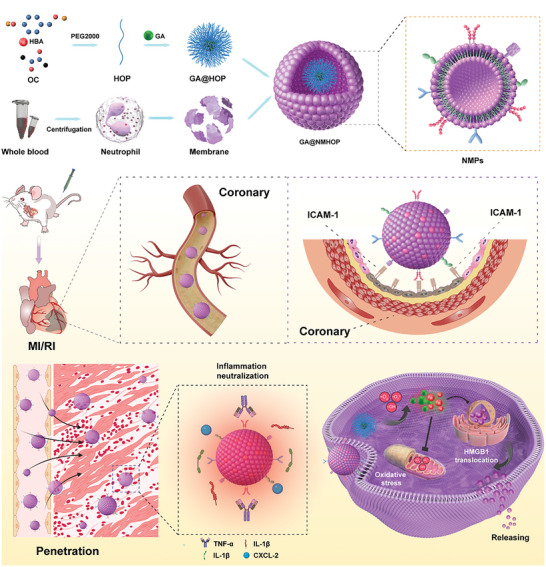
The schematic illustration depicts the fabrication process of NDs and the underlying principle of utilizing NDs to mitigate reperfusion injury in MI/RI by reprogramming the oxidative and inflammatory microenvironment. The excessive infiltration of immune cells and the subsequent generation of ROS contribute to heightened apoptosis of cardiomyocytes. Following intravenous injection into mice, NDs initially enter the bloodstream and subsequently accumulate within the injured heart. The NDs effectively deplete ROS levels and release GA, which hinders the nuclear translocation of HMGB1 and exerts a comprehensive inhibition of inflammatory cascades.

The use of cell membrane‐coating technology has emerged as a versatile and robust therapeutic platform, capable of neutralizing various pathogenic molecules and interacting with the surrounding environment.^[^ ^24^, ^25^ ^]^ These nanoparticles (NPs) are engineered by merging natural biological membranes with artificial cores, thereby inheriting the distinct antigenic profile of their parent cells and replicating essential cellular functions.^[^ ^26^ ^]^ Neutrophils, a key subset of immune cells, play a critical role in initiating inflammation and promoting wound healing at the site of cardiac ischemia caused by disrupted blood flow.^[^ ^27^, ^28^ ^]^ Our previous research demonstrated that neutrophil membrane‐coated NPs can accumulate in myocardial injury areas and neutralize various inflammatory mediators.^[^ ^29^ ^]^ In this study, we introduce an innovative neutrophil decoy (ND) specifically crafted to alleviate oxidative stress and suppress the inflammatory storm in the injured heart (**Scheme** **1**). We achieved this by chemically synthesizing p‐hydroxybenzyl alcohol (HBA), oxalyl chloride (OC), and PEG2000 into the ROS‐responsive amphiphilic copolymer HBA‐OC‐PEG2000 (HOP). Subsequently, 18β‐GA was loaded into the HOP NPs, which were then fused with membrane vesicles (MVs) derived from activated and purified mouse peripheral blood neutrophils. The outer‐layer neutrophil membrane shell enables the NDs to neutralize various cytokines, thus disrupting neutrophil infiltration and inflammation initiation. The ROS‐responsive core of NDs consumes intracellular ROS while releasing its cargos (HBA and 18β‐GA) to reprogram the inflammatory and oxidative microenvironment of MI/RI.

## 2 Results

### 2.1 Fabrication and Characterization of NDs

The synthesis of HOP was achieved through an OC‐assisted singular condensation polymerization process. The resulting copolyoxalate was subjected to sequential precipitations in chilled diethyl ether. After vacuum‐drying under amplified vacuum conditions, a pale yellow, transparent colloidal solid was obtained. The structural authenticity of HOP was later confirmed through ^1^H NMR spectroscopic analysis (Figure S1, Supporting Information).

**Figure 1:**
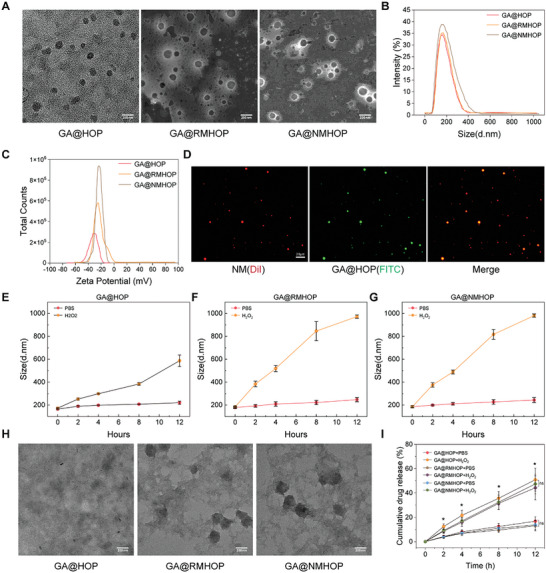
Characterizations of neutrophil membrane‐coated NPs. A) Representative TEM images of GA@HOP, GA@RMHOP, and GA@NMHOP. Scale bar = 200 nm. B) Hydrodynamic size distribution and C) ζ potential of GA@HOP, GA@RMHOP, and GA@NMHOP. D) Representative LSCM images of GA@NMHOP (red: neutrophil membrane labeled with DiI; green: GA@HOP labeled with FITC). Scale bar = 20 µm. E–G) Changes of NPs diameters over time (0, 2, 4, 8, 12 h) in the presence of H2O2. *n* = 3. H) Representative TEM images of GA@HOP, GA@RMHOP, and GA@NMHOP fragments after degradation in the presence of H2O2 for 12 h. Scale bar = 200 nm. I) Accumulated GA release from GA@HOP, GA@RMHOP, and GA@NMHOP in PBS or in the presence of 1 mM H2O2. *n* = 3. *p < 0.05 versus PBS group. All data are shown as means ± SD. Statistical analyses were performed by two‐tailed unpaired Student's t‐test and one‐way ANOVA followed by Tukey's post‐hoc test.

The encapsulation of 18β‐GA, referred to as GA@HOP, was accomplished using dialysis as the chosen method.^[^ ^30^ ^]^ NMVs were derived by isolating and purifying activated murine peripheral blood neutrophils. Subsequently, various NP formulations, including red blood cell membrane‐wrapped GA@RMHOP and neutrophil membrane‐wrapped GA@NMHOP, were crafted via repeated extrusion through polycarbonate porous membranes. TEM investigations confirmed the consistent and monodispersed core‐shell structure of the enveloped NPs (**Figure** **1**A). GA@RMHOP and GA@NMHOP displayed average diameters of 105.9 nm and 101.2 nm, respectively, while the unadorned GA@HOP lacked a distinct core‐shell morphology, with an average diameter of 100.9 nm (Figure S2A–C, Supporting Information). The hydrodynamic diameters of GA@HOP, GA@RMHOP, and GA@NMHOP were calculated to be 158.1 nm, 196.2 nm, and 185.1 nm, respectively, through DLS analysis (Figure 1B). The ζ potential values of GA@HOP, GA@RMHOP, and GA@NMHOP were identified as −31.6 mV, −22.9 mV, and −23.9 mV, respectively (Figure 1C), indicating successful membrane relocation onto the surface of GA@HOP NPs. This was further validated by the co‐localization of Dil‐labeled neutrophil membrane (red) and FITC‐labeled GA@HOP (green) as observed via Laser Confocal Microscopy, supporting the previous conclusion (Figure 1D).

Additionally, the DLC% of GA in GA@HOP, GA@RMHOP, and GA@NMHOP were measured at 7.91%, 5.7%, and 5.4%, respectively. The EE% of GA in these nanoparticles was determined to be 72%, 68.4%, and 69.1%, respectively. These findings confirm the efficient encapsulation and loading of GA within the nanoparticles, highlighting their potential for effective drug delivery. Digital imagery demonstrated the enduring stability of GA@HOP stored at 4 °C in PBS. The results showed that GA@HOP maintained prolonged stability for over 7 days, with no significant aggregation or decomposition, which are crucial for preservation and transportation (Figure S2D, Supporting Information).

Further investigation explored the potential inheritance of receptors for inflammatory factors and chemokines from the “parental” activated neutrophils by the NDs. SDS‐PAGE electrophoresis provided evidence that the primary protein constituents of the neutrophil membrane were preserved within GA@NMHOP (Figure S2E, Supporting Information). Similar to neutrophil lysate (NL) and NMVs, the NDs also displayed receptors for inflammatory factors, such as TNF‐αR (affinity to TNF‐α), interleukin 6 receptor (IL‐6R, affinity to IL‐6), and interleukin 1 receptor (IL‐1R, affinity to IL‐1β). In addition, the NDs expressed chemokine receptors such as CXCR1 (interacting with CINC‐1, CINC‐3, and LIX), CXCR2 (binding with CINC‐1, CINC‐2, CINC‐3, and LIX), along with lymphocyte function associated antigen‐1 (LFA‐1, which aids in the adhesion and rolling of neutrophils), and P‐selectin glycoprotein ligand‐1 (PSGL‐1, another facilitator of neutrophil adhesion and rolling). We also conducted three independent fabrications of NDs and evaluated the receptors present on NDs. As shown in Figure S2F (Supporting Information), the three independently prepared NDs displayed similar protein adsorption on their surfaces, and the key receptors were consistently retained in each replication. Therefore, these findings confirm the successful incorporation of neutrophil membrane proteins (NMPs) into NDs.

### 2.2 H_2_O_2_‐Responsive GA Release

Cells sustain damage upon ischemic injury or I/R insult, leading to the emission of ROS.^[^ ^30^ ^]^ The strategic design of controlled‐release nanocarriers offers a promising way to mitigate drug‐associated side effects, often stemming from off‐target impacts.^[^ ^31^ ^]^ To fully assess the H_2_O_2_‐responsive behavior, we examined the hydrolysis profiles of GA@HOP, GA@RMHOP, and GA@NMHOP within a pH 7.4 PBS solution, both with and without 1 mM H_2_O_2_. Over a 12‐h period at room temperature, the NPs showcased impressive stability, as evidenced by their unchanging size, in the absence of H_2_O_2_. In contrast, the introduction of H_2_O_2_ led to significant changes in particle size (Figure 1E–G). Furthermore, TEM image analysis showed the absence of stable structures in GA@HOP, GA@RMHOP, and GA@NMHOP after a 12‐h co‐incubation with H_2_O_2_ (Figure 1H). Quantitative analysis revealed that ≈12.41% of GA was released into 1 mM H_2_O_2_‐containing PBS after 2 h. Following this, the concentration of GA showed a time‐dependent increase, reaching a peak of 50.92% release at the 12‐h mark (Figure 1I). This release rate was markedly higher and swifter compared to the non‐H_2_O_2_ group.

### 2.3 Neutralization of Inflammatory Factors and Chemokines In Vitro

The orchestration of neutrophil recruitment in MI/RI necessitates intricate cytokine‐receptor coordination.^[^ ^32^ ^]^ However, targeting specific cytokines using antibodies or nanoscale particulates may be impeded by alternative cytokine surrogate functions. In contrast, NDs act as cytokine decoys, exhibiting superior efficacy in obstructing neutrophil recruitment and activation due to their diverse receptor repertoire.^[^ ^33^ ^]^ Our study aimed to evaluate the ability of NDs to adsorb inflammatory factors and chemokines in in vitro models. Different concentrations of RDs and NDs were incubated with TNF‐α, IL‐1β, IL‐6, and CXCL2. Following centrifugation, the remaining unconjugated cytokines were measured using ELISA kits. The residual cytokine fractions displayed a declining pattern with increasing ND concentrations, indicating significant adsorption capability of NDs in a concentration‐dependent manner (Figure S3A, Supporting Information). The half‐maximal inhibitory concentrations (IC50) were found to be 194.9 µg mL^⁻1^ for TNF‐α, 121.4 µg mL^⁻1^ for IL‐1β, 319.0 µg mL^⁻1^ for IL‐6, and 305.3 µg mL^⁻1^ for CXCL2 (Figure S3B, Supporting Information). Conversely, the RDs treatment group did not exhibit this concentration‐dependent neutralization trend (Figure S3C, Supporting Information).

**Figure 2:**
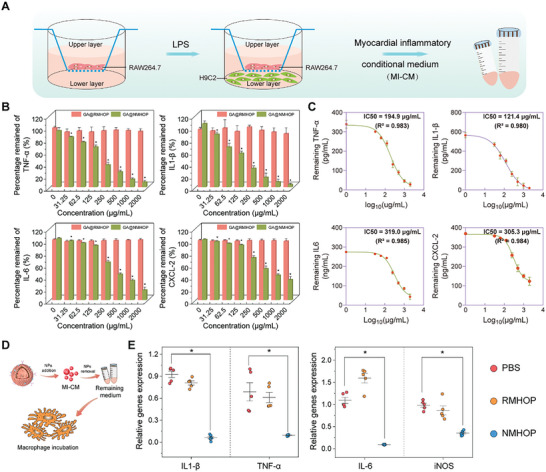
In vitro neutralization of inflammatory factors and chemokines. A) Schematic representation of the co‐culture of macrophages and cardiomyocytes. B) Dose‐response neutralization of proinflammatory factors, including TNF‐α, IL‐1β, IL‐6, and CXCL2, by both RDs and NDs in MI‐CM. *n* = 4. C) Binding capacity of NDs to inflammatory cytokines TNF‐α, IL‐1β, IL‐6, and CXCL2. IC50 values were determined using the variable slope model in GraphPad Prism 7. *n* = 4. D) Schematic illustration of RAW264.7 cell stimulation using MI‐CM. E) Analysis of RAW264.7 cell activation by measuring the relative mRNA expression levels of TNF‐α, IL‐1β, IL‐6, and iNOS through RT‐qPCR analysis. *n* = 5. *p < 0.05 versus PBS group. All data are shown as means ± SD. Statistical analyses were performed by one‐way ANOVA followed by Tukey's post‐hoc test.

To further substantiate the neutralization efficacy of NDs, an in vitro model simulating myocardial inflammation post I/R was established (**Figure** **2**A). This model exhibited significant elevation in inflammatory cytokine levels (TNF‐α, IL‐1β, IL6, and CXCL2). Upon co‐incubation with various concentrations of RDs and NDs, the NDs group once again demonstrated a clear pattern of concentration‐dependent neutralization of inflammatory factors (Figure 2B,C; Figure S3D, Supporting Information). Collectively, these empirical findings corroborate the capacity of NDs to adsorb inflammatory mediators.

The dynamic interaction of macrophage‐associated immune responses is of paramount importance in the context of MI/RI. The proinflammatory M1 macrophage phenotype plays a pivotal role in exacerbating cardiomyocyte injury.^[^ ^34^ ^]^ By harnessing the cytokine decoy properties of NDs, we evaluated their potential to mitigate macrophage activation. The MI‐CM, which contains inflammatory factors, was pre‐incubated with NDs for 2 h. Subsequently, the NDs were removed by centrifugation, and the resulting supernatant was employed for the cultivation of RAW264.7 cells (Figure 2D). Assessment of macrophage activation status via RT‐qPCR revealed a decrease in the levels of key activation markers (specifically, IL‐1β, TNF‐α, IL‐6, and iNOS), highlighting the efficacy of neutrophil membrane coating in absorbing factors that activate macrophages (Figure 2E).

Collectively, these empirical findings underscore that GA@NMHOPs exhibit substantial cytokine neutralization capability, effectively sponging local cytokines such as TNF‐α, IL‐1β, and IL‐6 in the local microenvironment, attributed to the profusion of cytokine receptors on their surface. This local cytokine neutralization helps in mitigating the inflammatory response and protecting cardiomyocytes from further damage.

### 2.4 Endothelial Cells (ECs) Adhesion and Penetration

**Figure 3:**
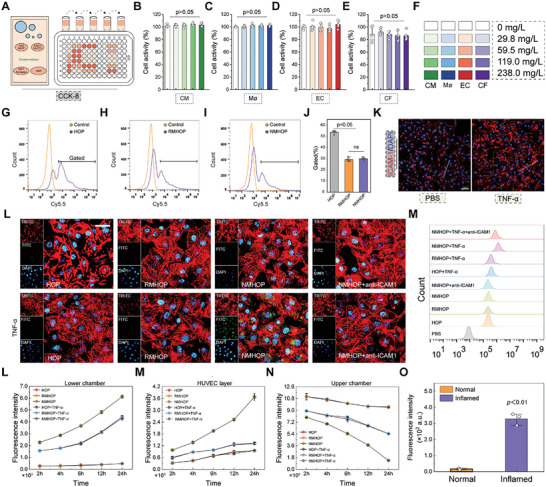
Targeting profile of NDs in vitro. A) Schematic diagram of CCK‐8. (B–F) Cell activity at five concentration gradients of NDs. B) CM: cardiomyocyte; C) MΦ: macrophage; D) EC: endothelial cell; E) CF: cardiac fibroblasts; F) Illustration of groups. *n* = 5. G–J) Uptake of GA@HOP, GA@RMHOP, and GA@NMHOP by macrophages in vitro. *n* = 6. K) Representative images of ICAM‐1 expression in HUVECs. Scale bar = 50 µm. L,M) Immunofluorescence and quantification of NPs conjugated with HUVECs. Scale bar = 20 µm. *n* = 3. L–N) Quantification of NPs distribution at different levels within the transwell system. *n* = 3. O) Quantitative analysis of fluorescence intensity in the lower chamber. *n* = 3. *p < 0.05 versus HOP group. All data are shown as means ± SD. Statistical analyses were performed by two‐tailed unpaired Student's t‐test and one‐way ANOVA followed by Tukey's post‐hoc test.

We subsequently evaluated the biosafety of GA@HOP across various cell types. At concentrations not exceeding 238.0 mg L^⁻1^ (GA: 18.8 mg L^⁻1^), cellular viability remained unimpaired, whether in cardiomyocytes, macrophages, ECs, or cardiac fibroblasts (**Figure** **3**A–F; Figure S4A–E, Supporting Information). To further examine the potential of NPs to evade immunosurveillance through membrane camouflage, HOP, RMHOP, or NMHOP were individually incubated with RAW264.7 cells for 2 h. Flow cytometry analysis revealed a marked reduction in macrophage clearance facilitated by the biomimetic membrane (Figure 3G–J).

Neutrophils participate in receptor‐mediated adhesion with cytokine‐activated ECs. Specifically, chemotaxis and adhesion between NMPs (primarily LFA‐1) and ICAM‐1 play a crucial role.^[^ ^35^ ^]^ Consequently, we investigated potential changes in ICAM‐1 expression on inflamed HUVECs. As depicted in Figure 3K and Figure S4F (Supporting Information), ICAM‐1 expression significantly upregulated upon TNF‐α stimulation of HUVECs. This upregulation was further confirmed through WB quantification. The protein levels of ICAM‐1 were significantly higher in TNF‐α treated HUVECs compared to untreated controls (Figure S4G,H, Supporting Information). And when HOP, RMHOP, and NMHOP were separately co‐incubated with HUVECs, NPs without membrane encapsulation or those encapsulated in red blood cell membranes exhibited negligible co‐labeling with HUVECs. In contrast, neutrophil membrane‐encapsulated NPs showed substantial binding to damaged HUVECs. Notably, this binding diminished significantly in normal HUVECs and in HUVECs where ICAM‐1 was blocked (Figure 3L,M). These results imply that the NMPs present on the NPs facilitates adhesion to damaged ECs, easing their subsequent movement across the endothelial layer into the injured heart area.

To determine the main process driving NPs crossing of the endothelial barrier, a transwell system was set up using HUVECs in the top compartment. Once 100% confluence was achieved, the medium was either treated with 100 ng mL^⁻1^ TNF‐α to replicate inflamed conditions in vivo or left untreated to mirror normal conditions. The results showed a significantly higher recovery of NMHOP from the lower chamber over time compared to HOP and RMHOP. This difference coincided with a relative reduction in the amount of NMHOP detected in the upper chamber. Moreover, NMHOP showed greater binding affinity to inflamed ECs compared to HOP and RMHOP. In the absence of damage to the ECs, the penetration of particles remained minimal (Figure 3L–N). These findings propose that NMHOP may traverse the endothelium primarily via either the enhanced permeability and retention (EPR) effect during inflammation or increased transcytosis. To differentiate between these two possibilities, HUVECs were exposed to NMHOP under inflamed conditions, both in the presence and absence of the endocytosis inhibitor dynasore (50 µM, 1 h) or the exocytosis inhibitor bafilomycin A1 (100 nM, 6 h). The results indicated that both dynasore and bafilomycin A1 only minimally impacted the transport of NPs across the endothelial barrier (Figure S4I, Supporting Information), suggesting that transcytosis was not the main route for NMHOP transmigration under inflamed conditions. Conversely, the use of fluorescence‐labeled 70 kDa dextran added to the upper chamber provided perspective on the permeability of the endothelium under both normal and inflamed circumstances. The findings demonstrated a notable augmentation of fluorescence intensity in the lower chamber after treatment with TNF‐α (Figure 3O), indicating that the permeability of the endothelial barrier was significantly enhanced. This increased permeability is a key factor facilitating NMHOP's ability to penetrate the endothelial layer. Collectively, these findings suggest that NMHOP primarily traverses between ECs through intercellular gaps rather than transcytosis (internalization followed by externalization).

### 2.5 Endocytosis and Protection of NDs

**Figure 4:**
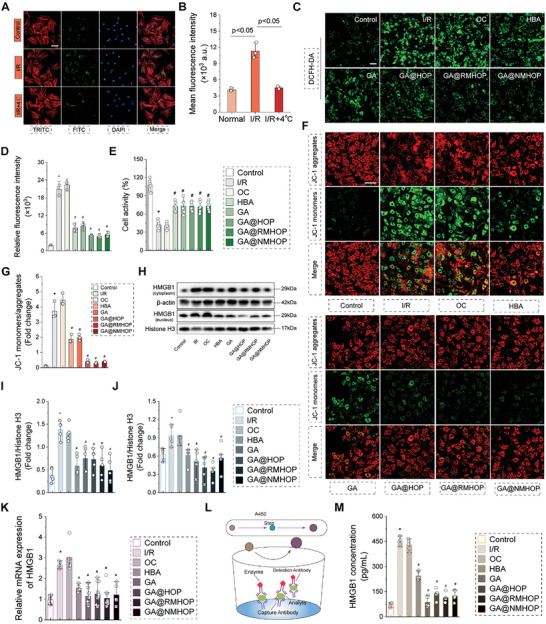
Protective effects of NDs in cardiomyocytes. A,B) Uptake assay and quantification of NDs by H9C2. Scale bar = 20 µm. *n* = 3. C) Representative images of DCFH‐DA fluorescence in H9C2 cells. Scale bar = 50 µm. D) Quantification of DCFH‐DA fluorescence intensity. *n* = 3. E) Cell activity of H9C2. *n* = 6. F) Representative images of JC‐1 (JC‐1 aggregate, red; JC‐1 monomer, green). Scale bar = 50 µm. G) Quantification of JC‐1 fluorescence intensity. *n* = 3. H) Western blot of HMGB1 in both the cytoplasm and nucleus. I,J) Quantification of HMGB1 in both the cytoplasm and nucleus. *n* = 5. K) The relative expression of HMGB1 mRNA. *n* = 5. L) Illustration of enzyme‐linked immunosorbent assay. M) Quantification of HMGB1 in H9C2 cell supernatants. *n* = 5. *p < 0.05 versus control group. #p < 0.05 versus I/R group. All data are shown as means ± SD. Statistical analyses were performed by one‐way ANOVA followed by Tukey's post‐hoc test.

The ability of NPs to penetrate cellular membranes and facilitate the release of therapeutic drugs is crucial in DDSs for intracellular regulation. Thus, we subsequently explored the uptake of GA@NMHOP within the microenvironment of MI/RI. Our results showed a significant increase in GA@NMHOP uptake under I/R conditions compared to the control group, suggesting that ischemic or reperfusion injury amplifies the endocytosis of GA@NMHOP by cardiomyocytes (Figure **4**A). This finding was further confirmed by quantitative analysis using flow cytometry (Figure 4B; Figure S5A–C, Supporting Information). Interestingly, we found that hypothermia (4 °C) effectively reversed the increased phagocytosis of GA@NMHOP, indicating an active process of internalization.

To better understand the specific mechanisms facilitating GA@NMHOP's entry into cardiomyocytes, we used various endocytosis inhibitors to block cellular internalization. Our experimental strategy included the use of chlorpromazine (CPZ, an inhibitor of clathrin‐mediated endocytosis), MβCD (an inhibitor of caveolae‐mediated endocytosis), and amiloride (an inhibitor of macropinocytosis). As shown in Figure S5D–G (Supporting Information), only the introduction of chlorpromazine significantly hindered the endocytosis of GA@NMHOP by cardiomyocytes. This suggests that neutrophil‐encapsulated NPs primarily penetrate the cell membrane via clathrin‐mediated endocytosis.

HBA, equipped with a phenolic hydroxyl group, exhibits a potent antioxidant capacity, effectively neutralizing hydroxyl radicals and superoxide.^[^ ^36^ ^]^ Within the intracellular environment, abundant in ROS, HBA is released due to peroxalate ester bond cleavage, thereby synergistically enhancing its antioxidant properties in tandem with GA. As delineated in Figure 4C,D and Figure S5H (Supporting Information), both HBA and GA have demonstrated their ability to counteract the surge of intracellular ROS in cardiomyocytes during I/R. The observed antioxidant activity can alleviate cell damage caused by I/R and protect cardiomyocytes (Figure 4E). Similarly, NPs encapsulating HBA and GA have shown comparable efficacy in ROS consumption. Mitochondrial dysfunction, a significant contributor to ROS production, is positively impacted by our DDS, which further curbed ROS generation by stabilizing the mitochondrial membrane potential through ROS sequestration (Figure 4F,G). While the differences between GA@NMHOP, GA@NMHOP, and GA@HOP were minimal, the similar antioxidant effects can be attributed to their comparable drug release rates and amounts in the in vitro ROS environment, rather than the type of cell membrane coating.

Extracellular HMGB1, acting as a potent innate “danger signal,” initiates host defense and tissue repair mechanisms. Before executing its functional role, HMGB1 necessitates translocation from the nucleus to the cytosol.^[^ ^37^ ^]^ To explore this translocation, we utilized WB analysis. As depicted in Figure 4H‐J, I/R not only augmented the nuclear abundance of HMGB1, but also facilitated its cytoplasmic translocation. However, cardiomyocytes treated with various GA formulations, including GA@HOP, GA@RMHOP, and GA@NMHOP, showed a significant reduction in both nuclear and cytoplasmic HMGB1 levels. Notably, HBA treatment, which is protective against I/R‐induced damage in cardiomyocytes, demonstrated similar effects. This protection is mediated by its ROS scavenging capacity, which, in turn, attenuates cytoplasmic translocation. The increase in HMGB1 content in the nucleus may be due to hypoxia‐induced upregulation of its transcription. To validate this hypothesis, we measured the levels of HMGB1 mRNA using RT‐qPCR. The results showed that hypoxia/reoxygenation increased the expression of the HMGB1 gene, while GA formulations partially reversed this effect (Figure 4K). Moreover, to corroborate these findings, we employed ELISA to measure the concentration of HMGB1 released into the cell supernatant (Figure 4L). The results affirmed that GA, GA@HOP, GA@RMHOP, and GA@NMHOP effectively curtailed the release of HMGB1 into the extracellular matrix (Figure 4M). In summary, the DDS we developed, loaded with GA, showcased its potential in curbing the expression of HMGB1 and its cytoplasmic translocation within cardiomyocytes. This, in turn, resulted in a decrease in the extrusion of HMGB1 into the surrounding extracellular environment.

### 2.6 Accumulation of NDs in Injured Hearts

**Figure 5:**
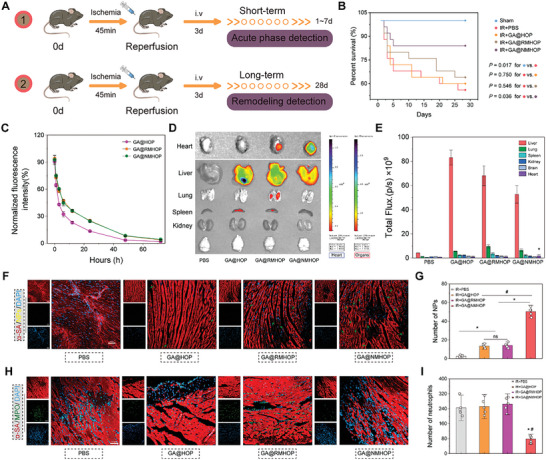
In vivo targeting profile of NDs. A) Schematic illustration showing the design of animal study. B) Survival curves of mice in each treatment group. C) Blood circulation profiles of GA@HOP, GA@RMHOP, and GA@NMHOP. *n* = 3. D) Distribution of GA@HOP, GA@RMHOP, and GA@NMHOP in different organs. E) Quantification of total fluorescence intensity of NPs in different organs. *n* = 3. F,G) Representative fluorescence microscopy images and quantification of NPs distribution in infarcted areas. *n* = 5. Scale bar = 50 µm. H,I) Representative fluorescence microscopy images and quantification of neutrophil infiltration in infarcted areas. *n* = 5. Scale bar = 50 µm. *p < 0.05 versus PBS group. #p < 0.05 versus GA@HOP, GA@RMHOP group. All data are shown as means ± SD. Statistical analyses were performed by one‐way ANOVA followed by Tukey's post‐hoc test.

To extend these in vitro observations into an in vivo context, we conducted both short‐ and long‐term experiments (**Figure** **5**A). Over the long term, the administration of GA@NMHOP significantly bolstered survival rates at 28 days post MI/R in mice (Figure 5B), underscoring the notable long‐term survival advantage of GA@NMHOP. In the short‐term in vivo studies, we first evaluated whether the encapsulation of cell membrane could prolong the systemic circulation of particles. As illustrated in Figure 5C, both GA@RMHOP and GA@NMHOP demonstrated extended circulation compared to GA@HOP, attributable to the protection afforded by biomimetic lipids as opposed to bare NPs.

Subsequently, we conducted an exhaustive assessment of the targeting ability of GA@NMHOP toward the inflamed myocardium. Key organs, including hearts, were retrieved 6 h following the injection of PBS, GA@HOP, GA@RMHOP, and GA@NMHOP. These were examined via macroscopic fluorescence imaging ex vivo, utilizing the IVIS system for visualization. As depicted in Figure 5D,E, GA@NMHOP treatment exhibited significantly higher heart accumulation compared to GA@HOP and GA@RMHOP treatments, driven by neutrophil chemotaxis. However, a significant proportion of NPs were distributed in the liver and spleen, which is similar with other NPs. Following this, the hearts were sectioned and stained with α‐SA, revealing that the majority of GA@NMHOP infiltrated the area surrounding the injured region of the hearts (Figure 5F,G). These observations suggest that GA@NMHOP has the capacity to target the injured myocardium and deliver 18β‐GA into cardiomyocytes, facilitated by its inflammatory tropism.

Furthermore, these pioneer particles can function as decoys, obstructing the subsequent infiltration of neutrophils into the injured myocardium and further thwarting the formation of an inflammatory storm after I/R (Figure 5H,I). Collectively, these data reveal the extended blood circulation, precise heart targeting, and decoy effect of GA@NMHOP under I/R conditions, thereby augmenting its therapeutic efficacy.

### 2.7 NDs Attenuated Infarct Size via Anti‐Inflammatory and Antioxidant Effects

**Figure 6:**
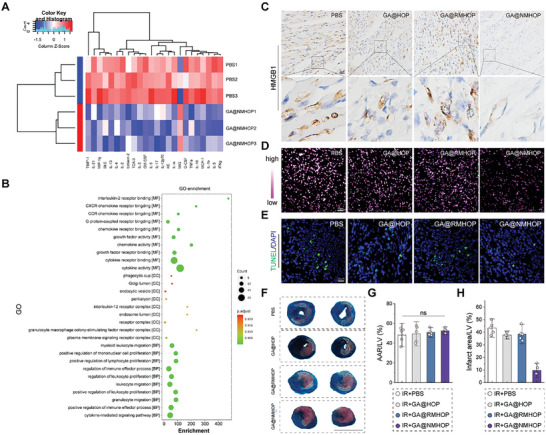
Inhibition of HMGB1 release and ROS production of cardiomyocytes. A) Heat map of inflammatory cytokines and chemokines. B) GO enrichment analysis of inflammatory cytokines and chemokines. C) Representative immunohistochemistry images of HMGB1 in myocardium. Scale bar = 50 µm. D) Representative images of DHE staining. Scale bar = 20 µm. E) Representative images of infarct size as stained by Evans Blue and TTC. Scale bar = 500 µm. F,G) Quantification of myocardial area at risk in relation to the left ventricle (AAR/V %) and infarct size (IS/AAR %). *n* = 5. H) TUNEL‐positive cardiomyocytes. Scale bar = 20 µm. *p < 0.05 versus PBS group. #p < 0.05 versus GA@HOP, GA@RMHOP group. All data are shown as means ± SD. Statistical analyses were performed by one‐way ANOVA followed by Tukey's post‐hoc test.

The inflammatory microenvironment remodeling by NDs was further investigated in the mouse model of MI/R. Expression levels of various cytokines and chemokines were assessed through microarray analysis. As shown in **Figure** **6**A,B, out of the 40 inflammatory cytokines targeted, 39 demonstrated a significant reduction. This suggests that NDs exert a broad‐spectrum anti‐inflammatory effect. The reduction in HMGB1 production and its extracellular release play a pivotal role in the anti‐inflammatory effect of NDs. Consequently, we next delved into the changes in expression and localization of HMGB1 in the cardiac tissue injury area using immunohistochemical methods. The results revealed that NDs can mitigate the elevated expression of HMGB1 induced by I/RI (Figure 6C; Figure S6A, Supporting Information).

Moreover, we found that NDs also display significant ROS‐scavenging abilities within the body (Figure 6D; Figure S6B, Supporting Information). The capacity of NDs to reshape the inflammatory microenvironment and efficiently eliminate ROS contributes to the reduction of cardiomyocyte apoptosis and the preservation of more endangered cardiomyocytes (Figure 6E; Figure S6C, Supporting Information). Further TTC/Evans blue staining disclosed that on the third day post I/R, despite similar sizes of areas at risk (AARs), the ratio of infarct area to AARs was significantly lower in NDs‐treated mice compared to other groups (Figure 6F–H). In summary, NDs can exert a beneficial cardiac protective effect by modulating inflammation and clearing ROS, thereby fostering a healthier cardiac function.

### 2.8 NDs Improve Ventricular Function and Limit Adverse LV Remodeling

**Figure 7:**
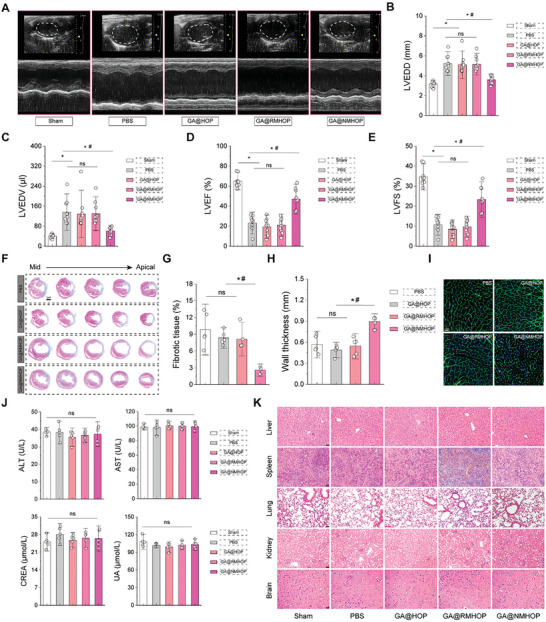
NDs improves ventricular function and limits adverse LV remodeling. A) Representative echocardiographic images on day 28. B–E) Quantitative analysis of LVEDD, LVEDV, LVEF (%) and LVFS (%) as assessed by echocardiography. *n* = 10. F) Representative Masson's trichrome staining. Scale bar = 1 cm. G,H) Quantification of fibrotic area and wall thickness. *n* = 5. I) Representative staining with WGA. Scale bar = 20 µm. J) Biochemical markers reflecting hepatic and renal function. *n* = 5. K) HE staining of important organs such as the brain, liver, spleen, kidney, and lung. Scale bar = 20 µm. *p < 0.05 versus Sham or PBS group. #p < 0.05 versus GA@HOP, GA@RMHOP group. All data are shown as means ± SD. Statistical analyses were performed by one‐way ANOVA followed by Tukey's post‐hoc test.

Following myocardial injury, the apoptosis of cardiomyocytes and fibrosis play pivotal roles in cardiac remodeling and eventual heart failure.^[^ ^38^ ^]^ We aimed to explore the potential of NDs‐based treatment to alleviate cardiac remodeling and maintain cardiac function. Echocardiography was employed on day 28 post‐I/R to assess cardiac functional parameters (**Figure** **7**A). Notably, the GA@NMHOP group exhibited a significant reduction in LV dilation, as evidenced by decreased LVEDD and LVEDV compared to the PBS group (Figure 7B,C). LVEF and FS assessments, reflecting systolic function, revealed enhanced values in MI/RI mice treated with GA@NMHOP (Figure 7D,E). These results imply improved systolic function and preserved the cardiac pumping function following GA@NMHOP administration.

We subsequently examined the effects of GA@NMHOP on LV remodeling. Masson's trichrome staining demonstrated a decrease in fibrotic tissue area and an increase in LV wall thickness in the GA@NMHOP group, surpassing other groups (Figure 7F–H). Analysis of the cardiomyocyte cross‐sectional area in the remote zone revealed a significantly smaller area in the GA@NMHOP group compared to other groups, suggesting improved cardiac function as well as mitigated adverse LV remodeling with GA@NMHOP treatment (Figure 7I; Figure S7, Supporting Information).

To further validate the in vivo biosafety of the NPs, we performed biochemical function assays to evaluate serum levels of ALT, AST, CREA, UA. These biomarkers, reflecting liver and kidney functions, exhibited no significant differences among groups (Figure 7J). Additionally, histopathological examination of major organ tissues revealed no discernible abnormalities, indicating the safety of GA@NMHOP at therapeutic dosages (Figure 7K).

## 3 Discussion

The cessation of blood flow, which interrupts the supply of oxygen and energy, followed by reperfusion, leading to a significant increase in ROS.^[^ ^6^ ^]^ These ROS cause severe cellular damage and trigger the release of danger‐associated molecular patterns (DAMPs), which subsequently activate immune defense and recruit inflammatory cells.^[^ ^39^ ^]^ This inflammatory response further amplifies ROS production, creating a harmful cycle.^[^ ^10^ ^]^ Traditional single‐target antioxidant or anti‐inflammatory treatments often fail to provide optimal therapeutic results because they only intervene at one stage of the inflammatory process. Therefore, simultaneous targeting of both ROS and inflammation could offer a more effective therapeutic approach. In this study, we underscore the potential of our nanodecoy system for treating MI/RI through several key innovations. First, we specifically target the oxidative stress and inflammation characteristic of MI/RI. Our advanced DDS combines GA with HOP, ensuring precise drug release under oxidative conditions. Second, by leveraging the unique properties of neutrophil membranes, we enhance adhesion and penetration to inflamed myocardial tissues. This targeting is further augmented by the therapeutic application of GA in cardiovascular diseases, an area with limited prior research. Finally, our system integrates antioxidant and anti‐inflammatory mechanisms, offering robust cellular protection and demonstrating significant in vivo efficacy, including reduced infarct size and improved cardiac function.

Modulating individual cytokines alone often proves inadequate in effectively intervening in the cascade of inflammatory responses due to multiple compensatory regulatory mechanisms within the inflammatory network.^[^ ^40^ ^]^ Similarly, directly scavenging and inhibiting multiple ROS to enhance antioxidant therapy effectiveness remains challenging with current treatment drugs or systems. In this study, we engineered a neutrophil‐based drug delivery nanodecoy with broad‐spectrum anti‐inflammatory and antioxidant capabilities. The DDS utilizes numerous membrane receptors on neutrophil surfaces to act as decoys for inflammatory factors, thereby attenuating the post‐MI/RI inflammatory storm. GA@NMHOP provides a multifaceted approach to combat MI/RI by sponging cytokines at the local inflammation site, reducing further damage. The ROS‐responsive release of GA and HBA provides targeted antioxidant effects, and the inhibition of HMGB1 translocation further mitigates inflammation. The high DLC% and EE% further ensure that GA@NMHOP can deliver an adequate amount of GA to the targeted site, maximizing its therapeutic efficacy while minimizing potential side effects. These combined effects significantly enhance myocardial recovery post‐MI/RI. Simultaneously, it inhibits the production and release of HMGB1, interrupting the further progression of the inflammatory cascade from its initial stage. Moreover, the HBA released in synergy with GA exerts both direct and indirect antioxidant regulatory effects. Ultimately, this system enables targeted and responsive drug release, improving treatment efficacy while minimizing potential side effects.

Extracellular HMGB1 acts as a potent innate “damage signal,” coordinating the assembly of inflammatory cells in response to acute damage. Specifically, in the context of I/R injury, HMGB1 plays a critical role in the early stages by binding to the receptor for advanced glycation end products (RAGE). This interaction triggers pro‐inflammatory signaling pathways, exacerbating myocardial damage.^[^ ^41^ ^]^ Targeting the HMGB1‐RAGE interaction has emerged as a novel therapeutic strategy for mitigating I/RI.^[^ ^37^ ^]^ Notably, GA has been shown to inhibit HMGB1 phosphorylation within cells, subsequently reducing its secretion. Furthermore, GA exhibits an inhibitory effect on overall HMGB1 expression.^[^ ^19^ ^]^ Thus, using GA to suppress HMGB1 expression and release can effectively mitigate the progression and severity of the inflammatory response during the initial stages of the cascade. Our findings confirm that GA administration significantly suppresses HMGB1 expression and prevents its translocation to the cytoplasm and extracellular matrix. This highlights the multifaceted benefits of GA, enabling intervention during the acute phase of inflammatory reactions and demonstrating comprehensive anti‐inflammatory efficacy, ultimately exerting broad‐spectrum inhibitory and therapeutic effects against inflammation. To validate this hypothesis, we performed a microarray analysis, which further confirmed the substantial reduction in the expression of 39 out of 40 examined inflammatory factors by GA.

HBA harnesses its antioxidative potential by utilizing its hydroxyl group to effectively scavenge hydroxyl radicals and superoxide species. Additionally, HBA can form peroxalate ester bonds with OC, enabling the responsive cleavage of ROS and subsequent release of both HBA and encapsulated GA. Similarly, when administered at appropriate concentrations, GA can upregulate various antioxidant enzymes, effectively inhibiting the generation of ROS.^[^ ^42^ ^]^ Our experimental findings indicate that the individual administration of HBA or GA leads to a reduction in intracellular ROS levels. Moreover, NPs incorporating both HBA and GA exhibit an enhanced antioxidative effect. This synergistic action of HBA and GA amplifies the pharmacological impact of the system, enabling a broader and more potent therapeutic approach. These findings highlight the potential of GA@NMHOP, GA@NMHOP, and GA@HOP as effective antioxidant therapies. The robust antioxidant capacities observed in all three formulations suggest that their similar drug release rates and amounts in the in vitro ROS environment play a critical role. The HOP ensures a consistent and controlled release of GA, which leads to comparable ROS scavenging effects across all formulations. Therefore, the type of cell membrane coating does not significantly influence the antioxidant properties in this in vitro setup. ROS‐responsive materials have the potential to restore ROS concentrations to normal levels, thereby reducing excessive oxidative stress and alleviating inflammation.^[^ ^43^ ^]^ Numerous moieties and linkers that respond to ROS have been engineered, contributing to the development of ROS‐responsive systems. These advancements facilitate the precise delivery of drugs and genes to their intended sites.^[^ ^44^ ^]^ Notably, recent reports have underscored the neuroprotective effects of GA‐conjugated polymeric NPs in the context of ischemic stroke.^[^ ^19^ ^]^ In light of these findings, a more rational strategy involves incorporating multiple drug components into our system, which synergistically combines targeted and penetrative properties for the treatment of myocardial injury.

Most current DDSs rely on modifications that target a single receptor or molecule. However, in myocardial tissue, particularly post‐I/R, there is a scarcity of highly abundant and specific target molecules. This scarcity makes it challenging for modifications that target a single molecule to achieve strong and precise targeting effects. Moreover, multiple barriers, such as the vascular wall and dense myocardial tissue, complicate the deep penetration of therapeutic systems into the heart tissue. Recently, cell‐membrane‐coated NPs have emerged as a promising therapeutic platform. Different cell types display a complex antigenic profile on their cell membranes, which contributes to their unique biological functions.^[^ ^45^ ^]^ Neutrophils, being the initial subset of immune cells reacting to cardiac damage, are quickly activated and attracted to the affected myocardium. Their presence peaks around the third day and persists, gradually accumulating over a period of 7 to 14 days following the onset of the injury.^[^ ^32^ ^]^ Neutrophil‐derived NPs, inheriting receptors from their parental neutrophils, effectively inhibit the recruitment and activation of neutrophils through multiple receptors on their surface. These receptors also offer a greater potential for interaction between neutrophil‐derived NPs and ECs.

For intravenous DDSs to achieve effective cardiac delivery, it is crucial that they successfully penetrate the intercellular gaps of ECs and the vascular wall barrier. Our findings suggest that NDs enhance their ability to cross ECs by strengthening their adhesion. This enhanced transendothelial interaction is primarily facilitated through the EPR effect, with a minor contribution from intracellular transport pathways. For drug molecules that require intracellular entry, increasing their accumulation within cells is crucial. Our study demonstrates that I/R injury can increase the entry of NDs into cardiomyocytes, primarily through clathrin‐mediated endocytosis. Unlike traditional single‐target treatments, GA@NMHOP offers a comprehensive approach by simultaneously addressing cytokine‐mediated inflammation, ROS‐induced oxidative stress, and HMGB1‐related pro‐inflammatory signaling. This multi‐target strategy provides a more effective means of reducing myocardial injury and promoting recovery.

Despite these promising results, this study has limitations. First, a longer follow‐up is needed to determine the long‐term therapeutic effects of NDs. Second, recent research by Wang et al. revealed the presence of a dense basement membrane barrier on the extravascular side of tumor blood vessels. This barrier severely impedes the extravasation capability of nanomedicines, resulting in their accumulation outside the tumor blood vessels, forming a “pool‐like” structure.^[^ ^46^ ^]^ Further investigation is required to determine if there are basement membrane‐like components outside the cardiac vasculature that impede the extravasation of nanomedicines. Third, the study did not elucidate how therapeutic strategies involving inflammation intervention impact the regeneration of blood vessels and cardiomyocytes after cardiac injury. Although various DDSs with targeting capabilities have been developed, a system that can achieve vascular penetration and deep infiltration into myocardial tissue, with benefits outweighing drawbacks, may be more clinically attractive.

## 4 Conclusions

To summarize, the results of our research show that meticulously constructed biomimetic targeting and ROS‐responsive neutrophil‐derived NPs can effectively instigate a reprograming of cardiac inflammation and oxidative stress. This was observed to be effective not only in controlled lab environments (in vitro) but also in living organisms (in vivo). This highly efficient targeting system possesses low immunogenicity and enhanced infiltrative capacity, while also exhibiting a broad spectrum of therapeutic effects. These advantages will significantly overcome the existing technological barriers that hinder clinical translation. Consequently, it will provide novel insights and intervention strategies for anti‐inflammatory and antioxidant therapies in MI/RI.

## 5 Experimental Section

### Synthesis of NP_s_

A chemical synthesis method was employed to prepare HOP.^[^ ^47^ ^]^ Briefly, in dry tetrahydrofuran (THF), HBA and OC were mixed and reacted under an ice bath for 2 h to form the HBA‐OC intermediate. PEG2000 was then added to the reaction system and allowed to react for an additional 4 h to form the HOP copolymer, with the reaction maintained at 4 °C. GA@HOP was synthesized using the nanoprecipitation method, where HOP and GA were individually dissolved in THF. The HOP THF solution was mixed with water, and then the appropriate amount of GA THF solution was added dropwise under sufficient stirring. The obtained mixture was subsequently dialyzed against water using a dialysis bag (MWCO 3.5 kDa) to remove any residual GA and THF.

### Neutrophil Collection

Neutrophils were isolated from the peripheral blood of 6‐week‐old male C57B/6 mice using the previously described Percoll gradient method.^[^ ^48^ ^]^ To activate neutrophils, mice were administered 1.5 mg k^−1^g lipopolysaccharide (LPS) intraperitoneally, and blood was collected 6 h later via submandibular bleeding. The uncoagulated blood was centrifuged (3000 × g, 5 minutes, 4 °C), and the top buffer layer was discarded. Gradients of 78%, 69%, and 52% (GE Healthcare, Chicago, USA) were prepared in a new tube before introducing the blood. After centrifugation at 1500 × g for 30 minutes, cellular contents between the interfaces of the 69% and 78% gradient layers, as well as those above the 78% gradient layer, were collected. Erythrocytes were lysed using a lysis buffer (Solarbio, Beijing, China) for 10 minutes on ice. The neutrophils were then gently washed three times with phosphate buffered saline (PBS) to remove any remaining contaminants. After washing, the neutrophils were stored in liquid nitrogen to preserve cell integrity, preparing them for subsequent membrane extraction.

### Cell Membrane Coating

Neutrophil membranes were effectively fused with NPs via extrusion. Briefly, after sonication, cell membrane fragments were combined with NPs. To ensure the effective coating of the NPs with the neutrophil membrane, a mass ratio of 1:2 (GA@HOP to neutrophil membrane) was used. The mixture was subsequently extruded ten times through a 200 nm polycarbonate porous membrane using an Avestin mini‐extruder (Avestin, Ottawa, Canada) to obtain membrane‐encapsulated NPs.

### NPs Characterization

Dynamic light scattering (DLS, Malvern, Herrenberg, Germany) was employed to measure the hydrodynamic sizes and surface zeta potentials of GA@HOP, GA@RMHOP, and GA@MHOP. The sample morphologies were analyzed using a transmission electron microscope (TEM, JEM‐1200EX, Jeol, Japan). The drug loading capacity (DLC%) and encapsulation efficiency (EE%) of GA in NPs were determined using high‐performance liquid chromatography (HPLC, Milford, MA, USA). The DLC% was calculated as (weight of GA in NPs / weight of NPs) × 100%, and the EE% was calculated as (actual drug content / theoretical drug content) × 100%. The presence of surface markers (PSGL‐1, LFA‐1, IL1R, IL6R, TNFα‐R, CXCR1, CXCR2) on neutrophils, neutrophil membranes, and neutrophil‐decorated NPs was confirmed via Western blotting (WB). An immunofluorescence study was performed to investigate membrane colocalization. For this purpose, NMVs were labeled with Dil, and GA@HOP was marked with FITC. GA@MHOP was visualized under a laser confocal microscope (Carl Zeiss LSM 880, Jena, Germany).

### In Vitro Drug Release

Hydrogen peroxide (H_2_O_2_)‐induced hydrolysis was investigated in PBS supplemented with 1 mM H_2_O_2_ at 37 °C. DLS was employed to monitor particle size changes at various time intervals (0, 2, 4, 8, and 12 h) during the hydrolysis process. Morphological changes were observed using TEM. To assess drug release, the supernatant was separated by centrifugation using a Millipore tube with a cutoff of 3.5 kDa, and the released drug was analyzed via HLPC.

### Inflammatory Factors and Chemokines Neutralization Assay

Recombinant mouse TNF‐α (0.5 ng mL⁻^1^), IL‐1β (1 ng mL⁻^1^), IL‐6 (0.7 ng mL⁻^1^), and CXCL‐2 (1 ng mL⁻^1^) were mixed with RMHOP and NMHOP at varying final concentrations (0 to 2 mg mL⁻^1^). Following a 2‐h incubation at 37 °C, RMHOP and NMHOP were removed, and the concentrations of remaining cytokines in the solution were detected using ELISA kits.

An in vitro myocardial inflammatory model was also established for the neutralization assay. Briefly, RAW264.7 cells (1 × 10^5^) were cultivated in the upper layer of the chambers, while H9C2 cells (1 × 10^5^) were cultured in the lower layer. RAW264.7 cells were starved for 24 h and then stimulated with LPS for 12 h. H9C2 cells were maintained in standard media. The culture medium from these two cell types was subsequently mixed in a 1:1 ratio to facilitate co‐culture. The myocardial inflammatory conditional medium (MI‐CM) was collected after 24 h, and the inflammatory factors in MI‐CM were quantified following incubation with NPs. Following a 2‐h incubation at 37 °C, RMHOP and NMHOP were removed, and the concentrations of remaining cytokines in the solution were detected using ELISA kits.

### In Vitro Targeting Assay

HUVECs (1 × 10^5^ cells/well) were cultured in a 6‐well culture plate with coverslips and incubated with 100 ng mL⁻^1^ TNF‐α for 12 h. FITC‐labeled HOP, RMHOP, and NMHOP were respectively introduced into the medium and incubated for 30 minutes at 4 °C. The cells were then washed with PBS, before getting fixed with a solution of 4% paraformaldehyde (PFA). Subsequently, they were stained with TRITC‐labeled phalloidin. Anti‐ICAM‐1 antibody was used for the control group. All samples were observed and quantified.

### WB

The WB analysis was performed according to the previous description.^[^ ^49^, ^50^ ^]^ Briefly, proteins were extracted from cells or tissues and quantified using a BCA kit (Beyotime, Shanghai, China). Following electrophoresis conducted under constant voltage conditions, the denatured proteins were transferred onto a polyvinylidene fluoride (PVDF) membrane. The membrane was then subjected to an overnight incubation at a temperature of 4 °C with primary antibodies, followed by a 2‐h incubation at room temperature with secondary antibodies. The protein bands were subsequently visualized using the GE AI600 imager (GE Healthcare, Buckinghamshire, UK).

### ROS Detection In Vitro

The H9C2 cells were placed in a hypoxia incubator for 6 h. Subsequently, the cells were transferred to a standard incubator with complete DMEM medium containing GA (18.8 mg L⁻^1^), HBA (11.4 mg L⁻^1^), OC (23.4 mg L⁻^1^), GA@HOP (238.0 mg L⁻^1^), GA@RMHOP (238.0 mg L⁻^1^), and GA@NMHOP (238.0 mg L⁻^1^) for 12 h. Cell viability was assessed using a CCK‐8 kit while intracellular ROS generation was measured with 2′,7′‐dichlorodihydrofluorescin diacetate (DCFH‐DA). The determination of inner membrane potential (ΔΨ_m) was conducted using JC‐1. Representative images were acquired, and the fluorescence intensities were quantified utilizing a microplate reader (SpectraMax M5, Molecular Devices, Sunnyvale, CA).

ROS detection in myocardial tissue was performed using Dihydroethidium (DHE) staining. Fresh myocardial tissue sections were incubated in a 5 µM DHE staining solution at room temperature for 30 minutes, protected from light. Following three washes with PBS, the sections were mounted with an anti‐fade reagent and examined under a confocal microscope for imaging and quantified by flow cytometry.

### Assay for HMGB1 Expression, Translocation, and Release In Vitro

H9C2 cells were exposed to hypoxia for 6 h. Following this, the culture medium was replaced with complete DMEM supplemented with GA, HBA, OC, GA@HOP, GA@RMHOP, and GA@NMHOP. The cells were then incubated for another 24 h in a standard incubator. The translocation of HMGB1 from the nucleus to the cytoplasm was assessed using WB. Briefly, nuclear and cytoplasmic proteins were extracted using a commercially available kit (Beyotime, Shanghai, China), and all samples underwent standard WB procedures. The concentration of extracellular HMGB1 was determined using an ELISA kit. The gene expression of HMGB1 was detected via Real‐Time PCR.

### Mouse Model of MI/RI and Administration

The animal procedures and postoperative care were conducted in accordance with the guidelines set by the National Institutes of Health and were approved by the Animal Care and Use Committee at Zhengzhou University (Approval No.ZZU‐LAC‐20230310). C57B/6 mice (Male, 7–8 weeks, 19–23 g) were housed in a controlled environment with a 12‐h light/dark cycle and had ad libitum access to water and food.

The MI/RI mouse models were induced according to the previously described method.^[^ ^51^ ^]^ In brief, mice were anesthetized with 2% isoflurane, and a left thoracic intercostal incision was made to expose the surgical field. To achieve a 45‐minutes ischemia, a 7‐0 nylon thread was passed under the lower border of the left descending coronary artery (LDA) and ligated. Five minutes before releasing the ligature, the mice were administered equal volumes of saline, GA@HOP (25 mg k^−1^g GA), GA@RMHOP (25 mg k^−1^g GA), or GA@NMHOP (25 mg k^−1^g GA) through the tail vein. The administration was repeated twice during the subsequent 48 h. In the sham group, the LDA was not ligated, and normal saline was used as the treatment.

### Pharmacokinetics, Biodistribution, and Biocompatibility

For pharmacokinetic analysis, mice were administered GA@HOP, GA@RMHOP, or GA@NMHOP intravenously at a dose equiv. to 25 mg k^−1^g of GA, five minutes before reperfusion. At predetermined intervals (0, 1, 3, 6, 12, 24, 48, and 72 h), 20 µL of whole blood were collected and centrifuged (800 × g, 10 minutes, 4 °C). The fluorescence intensity in the supernatant was measured using multifunctional microplate reader with an excitation wavelength of 673 nm and an emission wavelength of 707 nm.

For biodistribution analysis, a single dose of cy5.5‐labeled GA@HOP, GA@RMHOP, and GA@NMHOP was injected. Organs, including the heart, liver, lungs, spleen, kidneys, and brain, were collected at the 6‐h mark. Images were acquired using an in vivo imaging system (IVIS, PerkinElmer, Waltham, MA, USA) and assessed using Living Image 4.3.1 software.

To assess biocompatibility, blood samples were collected at 1 and 28 days post‐injection. The levels of aspartate aminotransferase (AST), alanine aminotransferase (ALT), creatinine (CREA), and blood urea nitrogen (BUN) in the serum of mice were analyzed using an automatic biochemical analyzer (Hitachi, Tokyo, Japan). The principal organs, including the heart, kidney, liver, spleen, lung, and brain, were subsequently fixed and subjected to hematoxylin and eosin (H&E) histological staining.

### Cardiac Function Evaluation

Under anesthesia, all animals received transthoracic echocardiograms using a VisualSonics Vevo 2100 Imaging System (VisualSonics, Toronto, ON, Canada) at 3 days and 4 weeks after MI/RI injury. The hearts were visualized in 2D from long‐axis perspectives at the level of the maximum left ventricular (LV) diameter. Subsequently, LV functional parameters, such as LV end‐diastolic volume (LVEDV), LV end‐diastolic dimension (LVEDD), LV shortening fraction (LVFS), and LV ejection fraction (LVEF), were calculated from representative views. Data were blinded and analyzed five times in random order.

### Histochemical and Immunohistochemical Analysis

The mice were humanely euthanized under deep anesthesia induced by isoflurane. The hearts were then collected and preserved in a suitable 4% PFA solution. To evaluate the extent of infarction and fibrosis, paraffin‐encased tissues were sectioned into 5–10 µm slices and subjected to Masson's trichrome staining. Both the total and fibrotic areas were quantified using ImageJ software. For neutrophil infiltration evaluation, freshly cryopreserved sections were restored to room temperature, rinsed with PBS, and incubated with an antigen‐retrieval solution (Solarbio, Beijing, China) for a duration of 20 min. Following a 30‐min blockade with an immunofluorescence blocking solution (Beyotime, Shanghai, China), the sections were incubated overnight with primary antibodies (anti‐α‐SA and anti‐MPO), followed by a 2‐h incubation with secondary antibodies (Alexa Fluor488, 594). Regions of interest were examined using a confocal microscope. Terminal deoxynucleotidyl transferase dUTP nick end labeling (TUNEL) staining was employed to determine the presence of apoptotic cardiomyocytes.

### Statistical Analysis

All data were presented as mean ± SD. A two‐tailed unpaired Student's t‐test was used for comparisons between the two groups. For comparisons among more than two groups, a one‐way ANOVA test was performed. *P* < 0.05 was considered statistically significant.

## Conflict of Interest

The authors declare no conflict of interest.

## Supporting information

Supporting Information

## Data Availability

The data that support the findings of this study are available from the corresponding author upon reasonable request.

## References

[1] M. D. Samsky , D. A. Morrow , A. G. Proudfoot , J. S. Hochman , H. Thiele , S. V. Rao , JAMA 2021, 326, 1840. 34751704 10.1001/jama.2021.18323 PMC9661446

[2] F. F. Gong , I. Vaitenas , S. C. Malaisrie , K. Maganti , JAMA Cardiol. 2021, 6, 341. 33295949 10.1001/jamacardio.2020.3690

[3] D. L. Bhatt , R. D. Lopes , R. A. Harrington , JAMA 2022, 327, 662. 35166796 10.1001/jama.2022.0358

[4] G. W. Reed , J. E. Rossi , C. P. Cannon , Lancet 2017, 389, 197. 27502078 10.1016/S0140-6736(16)30677-8

[5] M. S. Sabatine , E. Braunwald , J. Am. Coll. Cardiol. 2021, 77, 2822. 34082913 10.1016/j.jacc.2021.01.060

[6] M. Xiang , Y. Lu , L. Xin , J. Gao , C. Shang , Z. Jiang , H. Lin , X. Fang , Y. Qu , Y. Wang , Z. Shen , M. Zhao , X. Cui , Oxid. Med. Cell. Longevity 2021, 2021, 6614009. 10.1155/2021/6614009 PMC8149218 34055195

[7] M. Dambrova , C. J. Zuurbier , V. Borutaite , E. Liepinsh , M. Makrecka‐Kuka , Free Radical Biol. Med. 2021, 165, 24. 33484825 10.1016/j.freeradbiomed.2021.01.036

[8] Q. M. Chen , Trends Pharmacol. Sci. 2021, 42, 729. 34332753 10.1016/j.tips.2021.06.005 PMC8785681

[9] M. Sun , R. Wang , R. Xia , Z. Xia , Z. Wu , T. Wang , Front. Pharmacol. 2022, 13, 949754. 36120296 10.3389/fphar.2022.949754 PMC9470922

[10] F. He , L. Zuo , Int. J. Mol. Sci. 2015, 16, 27770. 26610475 10.3390/ijms161126059 PMC4661917

[11] Y. Yang , Y. Sun , W. Yi , Y. Li , C. Fan , Z. Xin , S. Jiang , S. Di , Y. Qu , R. J. Reiter , D. Yi , J. Pineal Res. 2014, 57, 357. 25230580 10.1111/jpi.12175

[12] F. Ruschitzka , J. S. Borer , H. Krum , A. J. Flammer , N. D. Yeomans , P. Libby , T. F. Luscher , D. H. Solomon , M. E. Husni , D. Y. Graham , D. A. Davey , L. M. Wisniewski , V. Menon , R. Fayyad , B. Beckerman , D. Iorga , A. M. Lincoff , S. E. Nissen , Eur. Heart J. 2017, 38, 3282. 29020251 10.1093/eurheartj/ehx508 PMC8139400

[13] R. A. Kloner , Circ. Res. 2013, 113, 451. 23908332 10.1161/CIRCRESAHA.112.300627

[14] P. A. Lalit , D. J. Hei , A. N. Raval , T. J. Kamp , Circ. Res. 2014, 114, 1328. 24723658 10.1161/CIRCRESAHA.114.300556 PMC4016859

[15] S. Wu , S. Cui , L. Wang , Y. Zhang , X. Yan , H. Lu , G. Xing , J. Ren , L. Gong , Acta Pharmacol. Sin. 2018, 39, 1865. 30061734 10.1038/s41401-018-0110-y PMC6289338

[16] P. Pan , Y. Wang , S. Lin , S. Liao , Y. Chen , W. Huang , C. Chen , W. Chen , Antioxidants 2022, 11, 961. 35624826 10.3390/antiox11050961 PMC9138139

[17] J. Jiang , X. Zhou , H. Chen , X. Wang , Y. Ruan , X. Liu , J. Ma , J Hazard Mater 2024, 471, 134319. 38657511 10.1016/j.jhazmat.2024.134319

[18] Z. Wang , J. Ma , Y. He , K. Miu , S. Yao , C. Tang , Y. Ye , G. Lin , Phytomedicine 2022, 102, 154162. 35598524 10.1016/j.phymed.2022.154162

[19] L. Jin , Z. Zhu , L. Hong , Z. Qian , F. Wang , Z. Mao , Bioact. Mater. 2023, 19, 38. 35415314 10.1016/j.bioactmat.2022.03.040 PMC8980441

[20] W. Quan , S. Kong , Q. Ouyang , J. Tao , S. Lu , Y. Huang , S. Li , H. Luo , Colloids Surf., B 2021, 205, 111791. 10.1016/j.colsurfb.2021.111791 34022703

[21] L. A. Stecanella , A. P. R. Bitencourt , G. R. Vaz , E. Quarta , J. O. C. Silva Júnior , A. Rossi , Pharmaceutics 2021, 13, 1792. 34834206 10.3390/pharmaceutics13111792 PMC8621092

[22] D. Wang , H. Wong , Y. Feng , Z. Zhang , J. Neuro‐Oncol. 2014, 116, 221. 10.1007/s11060-013-1292-2 24162829

[23] T. Kao , C. Wu , G. Yen , J. Agric. Food Chem. 2014, 62, 542. 24377378 10.1021/jf404939f

[24] R. H. Fang , A. V. Kroll , W. Gao , L. Zhang , Adv. Mater. 2018, 30, 1706759. 10.1002/adma.201706759 PMC5984176 29582476

[25] B. T. Luk , L. Zhang , J. Controlled Release 2015, 220, 600. 10.1016/j.jconrel.2015.07.019 PMC4688192 26210440

[26] J. Lopes , D. Lopes , M. Pereira‐Silva , D. Peixoto , F. Veiga , M. R. Hamblin , J. Conde , C. Corbo , E. N. Zare , M. Ashrafizadeh , F. R. Tay , C. Chen , R. F. Donnelly , X. Wang , P. Makvandi , A. C. Paiva‐Santos , Small Methods 2022, 6, e2200289. 35768282 10.1002/smtd.202200289

[27] C. SilvestreRoig , Q. Braster , A. OrtegaGomez , O. Soehnlein , Nat. Rev. Cardiol. 2020, 17, 327. 31996800 10.1038/s41569-019-0326-7

[28] S. D. Prabhu , N. G. Frangogiannis , Circ. Res. 2016, 119, 91. 27340270 10.1161/CIRCRESAHA.116.303577 PMC4922528

[29] D. Han , F. Wang , Z. Qiao , B. Wang , Y. Zhang , Q. Jiang , M. Liu , Y. Zhuang , Q. An , Y. Bai , J. Shangguan , J. Zhang , G. Liang , D. Shen , Bioact. Mater. 2023, 23, 369. 36474655 10.1016/j.bioactmat.2022.11.016 PMC9706603

[30] L. Jiang , X. Yin , Y. Chen , Y. Chen , W. Jiang , H. Zheng , F. Huang , B. Liu , W. Zhou , L. Qi , J. Li , Theranostics 2021, 11, 1703. 33408776 10.7150/thno.43895 PMC7778584

[31] S. Sharifi , G. Caracciolo , M. Mahmoudi , Trends Pharmacol. Sci. 2020, 41, 641. 32713606 10.1016/j.tips.2020.06.011

[32] G. F. Baxter , Basic Res. Cardiol. 2002, 97, 268. 12111036 10.1007/s00395-002-0366-7

[33] Y. Li , F. Lin , Acta Biomater. 2019, 99, 330. 31446047 10.1016/j.actbio.2019.08.033 PMC7066532

[34] C. Peet , A. Ivetic , D. I. Bromage , A. M. Shah , Cardiovasc. Res. 2020, 116, 1101. 31841135 10.1093/cvr/cvz336 PMC7177720

[35] N. G. Frangogiannis , Circ. Res. 2012, 110, 159. 22223212 10.1161/CIRCRESAHA.111.243162 PMC3690135

[36] S. Kim , H. Park , Y. Song , D. Hong , O. Kim , E. Jo , G. Khang , D. Lee , Biomaterials 2011, 32, 3021. 21292318 10.1016/j.biomaterials.2010.11.033

[37] M. Andrassy , H. C. Volz , J. C. Igwe , B. Funke , S. N. Eichberger , Z. Kaya , S. Buss , F. Autschbach , S. T. Pleger , I. K. Lukic , F. Bea , S. E. Hardt , P. M. Humpert , M. E. Bianchi , H. Mairbäurl , P. P. Nawroth , A. Remppis , H. A. Katus , A. Bierhaus , Circulation 2008, 117, 3216. 18574060 10.1161/CIRCULATIONAHA.108.769331

[38] D. P. Del Re , D. Amgalan , A. Linkermann , Q. Liu , R. N. Kitsis , Physiol. Rev. 2019, 99, 1765. 31364924 10.1152/physrev.00022.2018 PMC6890986

[39] F. Arslan , D. P. de Kleijn , G. Pasterkamp , Nat. Rev. Cardiol. 2011, 8, 292. 21448140 10.1038/nrcardio.2011.38

[40] Tedgui , Z. Mallat , Physiol. Rev. 2006, 86, 515. 16601268 10.1152/physrev.00024.2005

[41] E. Foglio , L. Pellegrini , M. A. Russo , F. Limana , Cells 2022, 11, 216. 35053332 10.3390/cells11020216 PMC8773872

[42] X. Ma , H. Chen , L. Cao , S. Zhao , C. Zhao , S. Yin , H. Hu , Phytother. Res. 2021, 35, 6932. 34709693 10.1002/ptr.7310

[43] H. Ye , Y. Zhou , X. Liu , Y. Chen , S. Duan , R. Zhu , Y. Liu , L. Yin , Biomacromolecules 2019, 20, 2441. 31117357 10.1021/acs.biomac.9b00628

[44] W. Chen , D. Li , Front. Chem. 2020, 8, 732. 32974285 10.3389/fchem.2020.00732 PMC7472733

[45] W. Liu , M. Zou , S. Qin , Y. Cheng , Y. Ma , Y. Sun , X. Zhang , Adv. Funct. Mater. 2020, 30, 2003559.

[46] Q. Wang , Q. Liang , J. Dou , H. Zhou , C. Zeng , H. Pan , Y. Shen , Q. Li , Y. Liu , D. T. Leong , W. Jiang , Y. Wang , Nat. Nanotechnol. 2023, 11, 216. 10.1038/s41565-023-01498-w 37709950

[47] L. Luo , G. Zang , B. Liu , X. Qin , Y. Zhang , Y. Chen , H. Zhang , W. Wu , G. Wang , Theranostics 2021, 11, 8043. 34335979 10.7150/thno.60785 PMC8315061

[48] T. Kang , Q. Zhu , D. Wei , J. Feng , J. Yao , T. Jiang , Q. Song , X. Wei , H. Chen , X. Gao , J. Chen , ACS Nano 2017, 11, 1397. 28075552 10.1021/acsnano.6b06477

[49] D. Han , F. Wang , B. Wang , Z. Qiao , X. Cui , Y. Zhang , Q. Jiang , M. Liu , J. Shangguan , X. Zheng , Y. Bai , C. Du , D. Shen , Front. Pharmacol. 2022, 13, 830763. 35185583 10.3389/fphar.2022.830763 PMC8850779

[50] D. Han , B. Wang , X. Cui , W. He , Y. zhang , Q. Jiang , F. Wang , Z. Liu , D. Shen , J. Cell. Mol. Med. 2021, 25, 1074.

[51] T. Su , K. Huang , H. Ma , H. Liang , P.‐U. Dinh , J. Chen , D. Shen , T. A. Allen , L. Qiao , Z. Li , S. Hu , J. Cores , B. N. Frame , A. T. Young , Q. Yin , J. Liu , L. Qian , T. G. Caranasos , Y. Brudno , F. S. Ligler , K. Cheng , Adv. Funct. Mater. 2019, 29, 1803567. 32256277 10.1002/adfm.201803567 PMC7111457

