# Supplementary material for: Enhancing Cardioprotection Through Neutrophil‐Mediated Delivery of 18β‐Glycyrrhetinic Acid in Myocardial Ischemia/Reperfusion Injury
Source: Adv Sci (Weinh). 2024 Sep 12;11(42):2406124. doi: 10.1002/advs.202406124 (PMC11558124; doi:10.1002/advs.202406124)
Supplement: Supplementary file 1 — Supporting Information [file ADVS-11-2406124-s001.docx]

**Supporting Information**

**Enhancing cardioprotection through neutrophil-mediated delivery of 18β-glycyrrhetinic acid in myocardial ischemia/reperfusion injury**

Dongjian Han^1,2,*,§^, Fuhang Wang^1,2,*^, Qingjiao Jiang^1,2^, Zhentao Qiao^3^, Yuansong Zhuang^1,2^, Quanxu An^1,2^, Yuhang Li^1,2^, Yazhe Tang^1,2^, Chenyao Li^1,2^, Deliang Shen^1,2,§^

^1^Department of Cardiology, The First Affiliated Hospital of Zhengzhou University, Zhengzhou, 450052, China

^2^Key Laboratory of Cardiac Injury and Repair of Henan Province, Zhengzhou, China

^3^Department of Vascular and Endovascular Surgery, The First Affiliated Hospital of Zhengzhou University, Zhengzhou, 450052, China

^§^Correspondence:

Deliang Shen

Address: No.1 East Construction Road, The First Affiliated Hospital of Zhengzhou University, Zhengzhou, 450052, China.

Phone: +86 18595871805

Email: dlshen@zzu.edu.cn (DL. Shen)

Dongjian Han

Address: No.1 East Construction Road, The First Affiliated Hospital of Zhengzhou University, Zhengzhou, 450052, China.

Phone: +86 18838281942

Email: hdj02012859@gs.zzu.edu.cn (DJ. Han)

*Equal contribution: These authors have equal contributions.

**Methods Section**

**Materials**

p-Hydroxybenzyl alcohol (HBA), oxalyl chloride (OC), and poly-(ethylene glycol)2000 (PEG2000), tetrahydrofuran (THF), hydrogen peroxide (H_2_O_2_, 30%), and trichloromethane were purchased from Aladdin Bio-Chem Technology Co, Ltd. (Shanghai, China). 18β-Glycyrrhetinic acid (GA), Dynasore, Bafilomycin A1, Chlorpromazine, Genistein, Cytochalasin D, Dichlorofluorescein diacetate (DCFH-DA), and lipopolysaccharide (LPS) were purchased from MedChemExpress (New Jersey, USA). D-mannitol, sucrose, EGTA, and a cocktail of protease, phosphatase inhibitors, and JC‐1 fluorescent probe were purchased from Thermo Fisher Scientific (MA, USA). Polyvinylidene fluoride (PVDF) membranes were purchased from Millipore (Billerica, MA, USA). CCK‐8 kits were purchased from Guangzhou Barley Biotechnology (China). Anti-TNFαR antibody, anti-LFA-1 antibody, anti-IL1R antibody, anti-PSGL-1 antibody, anti-IL6R antibody, and anti-αSA antibody were purchased from Abcam (Cambridge, USA). Anti-CXCR1 antibody and anti-CXCR2 antibody were purchased from HuaBio (Hangzhou, China). Anti-HMGB1 antibody, anti-β-actin antibody, anti-histone 3 antibody, and anti-MPO antibody were purchased from Cell Signaling Technology (Danvers, MA, USA).

**Cell membrane derivation**

The frozen cells were subjected to three washes with phosphate-buffered saline (PBS), followed by centrifugation at 800 g for 5 minutes. The cells were then suspended in a hypotonic lysing buffer containing 225 mM D-mannitol, 75 mM sucrose, and 0.2 mM EGTA, along with a cocktail of protease and phosphatase inhibitors. Cell disruption was achieved using a Dounce homogenizer, and the resulting homogenate was centrifuged at 20,000 × g for 25 minutes at 4 °C to remove mitochondria. The supernatant was subjected to further centrifugation at 100,000 g for 35 minutes at 4 °C, and the resulting membrane pellets were collected and washed twice with 0.2 mM EDTA in water. The membrane content was determined using a BCA kit (Beyotime, Shanghai, China). Following suspension in 0.2 mM EDTA, the membrane was stored at -80 °C for future utilization.

The generation of the red blood cell (RBC) membrane involved a series of sequential procedures. Initially, RBCs were suspended in 0.25 × PBS and kept in an ice bath for a duration of 20 minutes. Following this, the suspension underwent centrifugation (5 minutes, 800 × g) to eliminate hemoglobin. This centrifugation process was repeated until complete removal of hemoglobin was achieved. The resultant pink pellet, consisting of the RBC membrane, was quantified and subsequently stored at -80 °C for future application.

**Cell culture**

The Rat embryonic cardiomyoblast cell line (H9C2), macrophage (RAW264.7), and human umbilical vein endothelial cells (HUVECs) were purchased from the American Type Culture Collection (ATCC). HUVECs were cultured in human endothelial cell growth medium (ECM, Sciencell, CA, USA), while H9C2 cells and RAW 264.7 cells were grown in high-glucose Dulbecco's High Glucose Modified Eagles Medium (DMEM, VivaCell, Shanghai, China). Primary murine cardiac fibroblasts (mCFs) were obtained from iCellBioscience (Shanghai, China) and maintained in DMEM medium. All media were supplemented with 10% (v/v) FBS, penicillin (100 U/mL), and streptomycin (100 μg/mL). Cells were incubated in a humidified incubator (Thermo Scientific, MA, USA) at 37 °C with 5% CO_2_.

**In vitro cytotoxicity and cell uptake assays**

H9C2, RAW264.7, HUVEC cells, and cardiac fibroblasts were incubated with a series of concentrations of GA, GA@RMHOP, and GA@NMHOP for 24 hours. Cell activity was analyzed using a Cell Counting Kit-8 (CCK-8, Dojindo, Japan). For cell uptake assays, H9C2 cells were seeded in 6-well plates (1×10^5^/well), subjected to hypoxia for 6 hours, and then the medium was replaced with fresh DMEM containing FITC-labeled HOP, RMHOP, or NMHOP (18.8mg GA/L) and placed in a standard incubator for 2 hours.

To further investigate the mechanisms of uptake, H9C2 cells were subjected to hypoxia for 6 hours, then incubated with 10 μg/mL chlorpromazine (clathrin-mediated endocytosis inhibitor), 5 mg/mL Methyl-β-cyclodextrin (MβCD, caveolae-mediated endocytosis inhibitor), 10 μg/mL amiloride (macropinocytosis inhibitor), for a period of 2 hours each. Following this, the medium was replaced with fresh DMEM containing NMHOP (with a concentration of 18.8 mg GA/L) and incubated in a standard incubator at a temperature of 37 ℃ for 2 hours. The uptake efficiency was quantitatively analyzed using flow cytometry (ACEA Biosciences Inc., San Diego, CA, USA).

**Macrophages activation assay**

To further assess the influence of RMHOP and NMHOP on the activation of RAW264.7 cells, RMHOP (500 μg/mL) or NMHOP (500 μg/mL) were pre-incubated with MI-CM. After centrifugation, the pre-incubated MI-CM or control medium was added to the medium to induce activation of RAW264.7 cells for 24 hours. Subsequently, a reverse transcriptase-quantitative polymerase chain reaction (RT-qPCR) assay was conducted on the RNA isolated from the cells using Trizol® Reagent (Invitrogen, CA, USA) to evaluate the expression of TNF-α, IL-1β, IL-6, and iNOS genes.

**Transwell assay**

In order to investigate the effectiveness of nanoparticle passage through an inflamed heart in a controlled laboratory setting, a capillary intimal barrier model was established using human umbilical vein endothelial cells (HUVECs) and a transwell cell culture system. Specifically, HUVECs (1×10^5^ cells/well) were seeded in the upper chamber (0.4 μm) and activated with TNF-α (200 ng/mL) for 12 hours. Subsequently, a fresh medium containing FITC-labeled HOP, RMHOP, and NMHOP was introduced for varying time intervals (2, 4, 6, 12, and 24 hours) to allow for nanoparticle crossing. The fluorescence intensities in the upper, intracellular, and lower chambers were measured using a microplate reader (Spectramax M5, Molecular Devices, CA, USA).

**RT-qPCR**

Total RNA was extracted from cells or tissues and reverse transcribed into complementary DNA (cDNA). Real-Time PCR was performed to detect the expression of the target gene. The primers are summarized in Table S2. Gene expression was normalized to the internal control gene GAPDH expression.

**TTC/Evans Blue Staining**

To assess myocardial infarct size, TTC/Evans blue staining was performed as follows. After reperfusion, the hearts were excised and immediately perfused with 1% Evans blue dye via the aorta to delineate the area at risk (AAR). The hearts were then sliced into 1 mm sections and incubated in 2% 2,3,5-triphenyltetrazolium chloride (TTC) solution at 37°C for 20 minutes. TTC stains viable myocardium red, while infarcted areas remain pale. The sections were fixed in 4% paraformaldehyde and photographed. Image analysis was performed using ImageJ software to calculate the infarct size as a percentage of the AAR.

**Protein microarray of inflammatory cytokines and chemokines**

Fresh myocardial tissues were obtained three days following myocardial ischemia/reperfusion injury (MI/RI) and thoroughly pulverized. One hundred microliters of the resulting tissue supernatant were utilized for the detection and quantification of cytokines and chemokines using the Quantibody Mouse Inflammation Array I kit (RayBiotech, Inc. Norcross, GA), following the manufacturer's instructions. The signals were recorded using a GenePix 4000B laser scanner (Bio-Rad Laboratories, Hercules, CA) and analyzed using GenePix Pro 6.0 microarray analysis software. The RayBiotech Mouse Inflammation Array 1 software (QAM-INF-1_Q-Analyzer) was utilized for conducting quantitative data analysis.

**Results Section**

**Table S1. Contents of figures**

| **Figures** | **Titles** |
| --- | --- |
| **Figure S1** | The NMR results of HOP. |
| **Figure S2** | The characterizations of fabricated NPs. |
| **Figure S3** | Neutralization assay of RDs and NDs. |
| **Figure S4** | Safety and targeting evaluation of NPs in vitro. |
| **Figure S5** | Uptake of NDs in Cardiomyocytes. |
| **Figure S6** | Antioxidative and anti-inflammatory activities of NDs. |
| **Figure S7** | Quantitative analysis of myocyte cross-sectional area. |

**
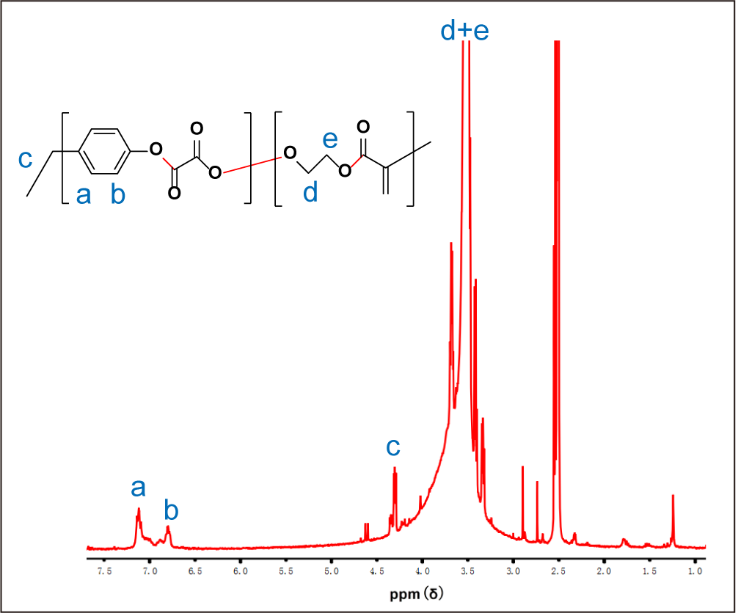
**

**Fig. S1.** The NMR results of HOP. The confirmation of the chemical structure of HOP was achieved through ^1^H NMR analysis in deuterated DMSO-*d*_6_ on a 400 MHz spectrometer. By comparing the resonance signal of the integrated methoxy group of the PEG2000 chain at approximately 3.5 ppm, it was observed that the resonance peaks at around 4.3 ppm correspond to the methylene protons adjacent to oxalate ester linkages. Additionally, two multiplet aromatic proton peaks were observed at approximately 6.8 and 7.2 ppm. These findings provide evidence for the successful polymerization resulting from the condensation reaction between OC, PEG2000, and HBA, leading to the formation of polyoxalate containing peroxalate ester linkages.

| **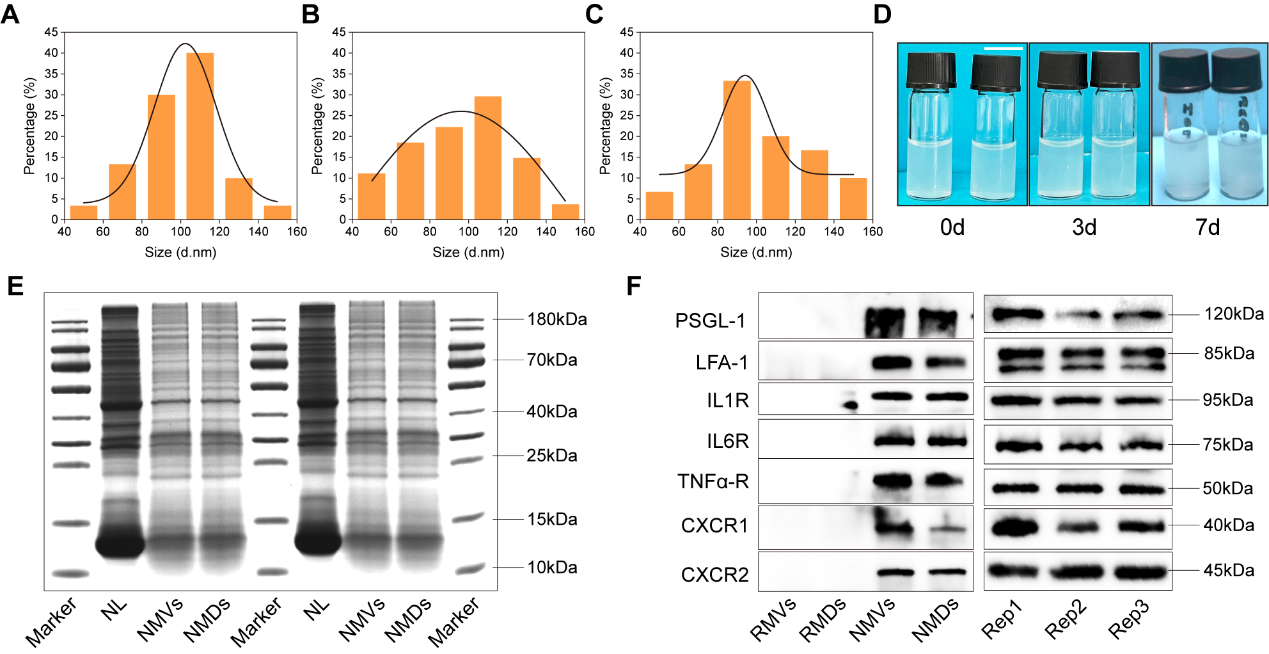** |
| --- |
| **Fig. S2.** The characterizations of fabricated NPs. (A-C) The diameter distribution of GA@HOP, GA@RMHOP, and GA@NMHOP measured by TEM. (D) Digital photos of HOP (left bottle) and GA@HOP (right bottle) in saline at 4 °C for a series of time. Scale bar = 1 cm. (E) SDS-PAGE protein analysis of NL, NMVs, and NMDs. NL: neutrophil lysate. (F) Immunoblotting for membrane receptors including PSGL-1, LFA-1, IL1R, IL6R, TNFα-R, CXCR1, and CXCR2. RMVs: membrane vesicles of red blood cells; RMDs: red blood cell decoys; NMDs: neutrophil decoys; NMVs: membrane vesicles of neutrophil. n = 3. All data are shown as means ± SD. |

| **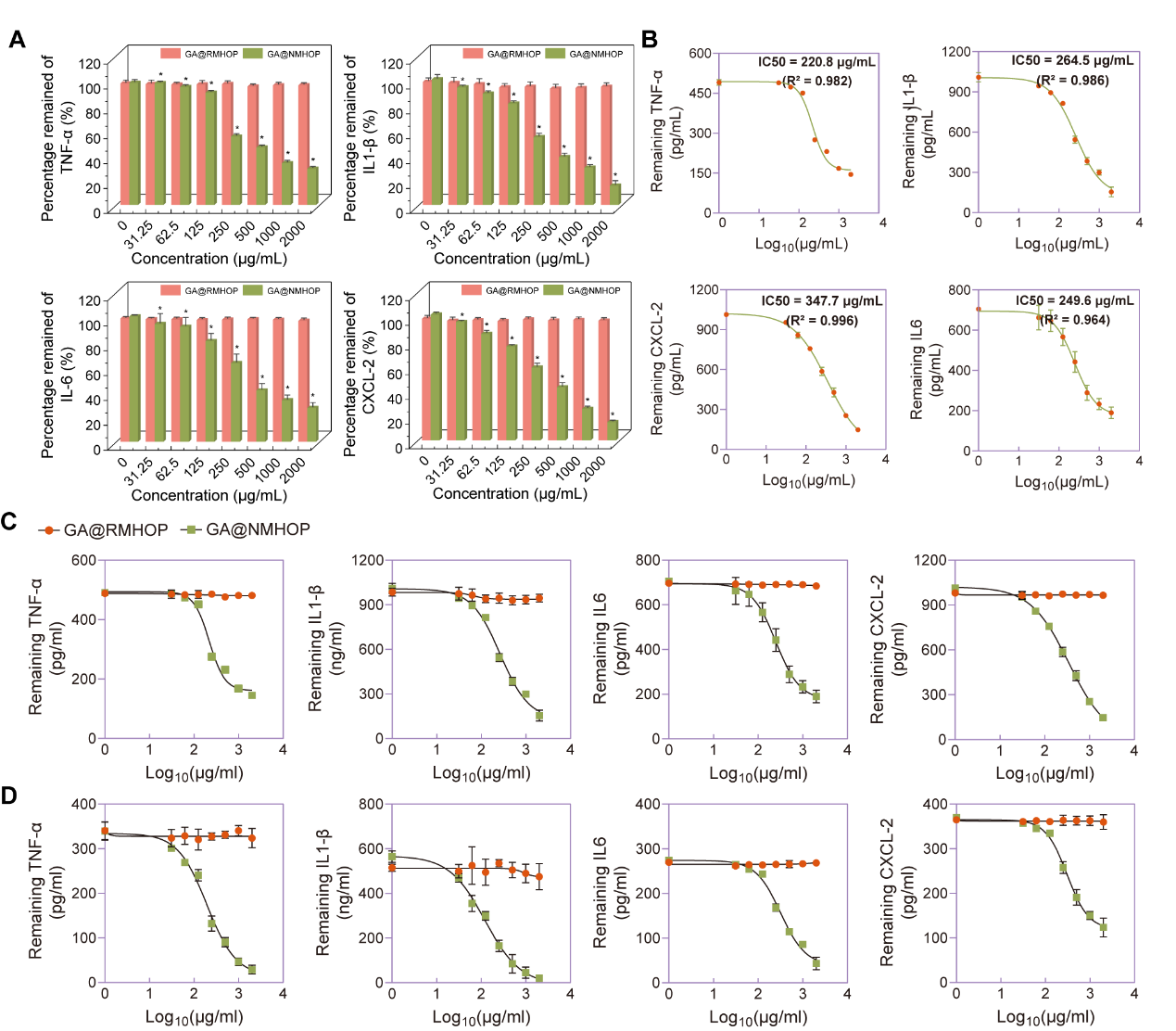** |
| --- |
| **Fig. S3.** Neutralization assay of RDs and NDs. (A) The adsorption capacity of RDs and NDs to inflammatory factors including TNF-α, IL-1β, IL-6, and CXCL2. n = 4. (B) Binding capacity of NDs to inflammatory cytokines, namely TNF-α, IL-1β, IL-6, and CXCL2. n = 4. (C) Binding capacity of RDs and NDs to inflammatory cytokines, namely TNF-α, IL-1β, IL-6, and CXCL2. n = 4. (D) Binding capacity of RDs and NDs to inflammatory cytokines, namely TNFα, IL1β, IL6, and CXCL2 in MI-CM. n = 4. ^*^*p* < 0.05 vs. PBS group. All data are shown as means ± SD. Statistical analyses were performed by one-way ANOVA followed by Tukey’s post-hoc test. |

| **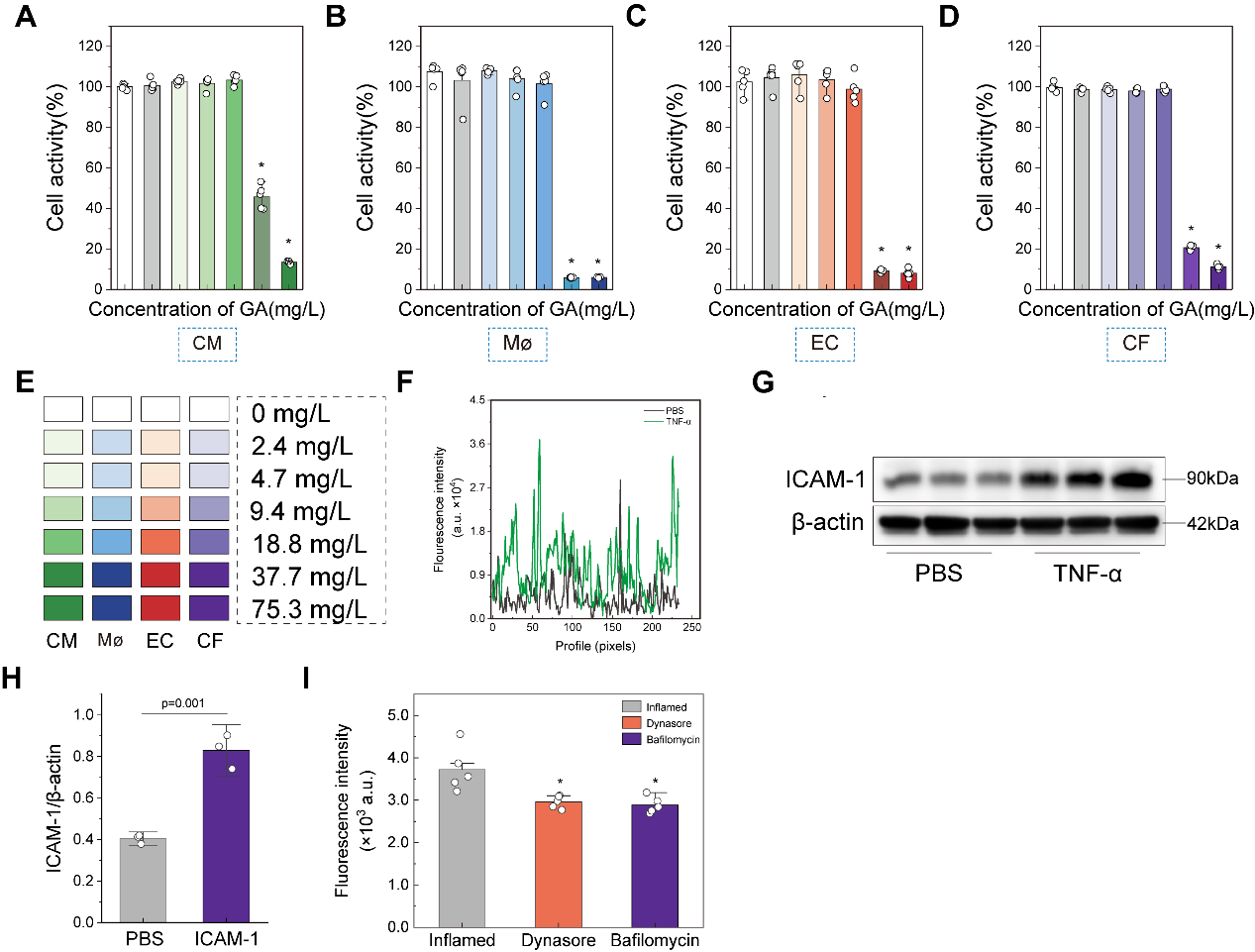** |
| --- |
| **Fig. S4.** Safety and targeting evaluation of NPs in vitro. (A-E) Cell activity was assessed with the CCK-8 assay kit. n = 5. (F) Quantification of the fluorescence intensity of ICAM-1. n = 3. (G-H) ICAM-1 expression detecting by WB and quantification. (I) Quantitative analysis of fluorescence intensity in the lower chamber. n = 5. ^*^*p* < 0.05 vs. PBS group. All data are shown as means ± SD. Statistical analyses were performed by Student's t-test and one-way ANOVA followed by Tukey’s post-hoc test. |

| **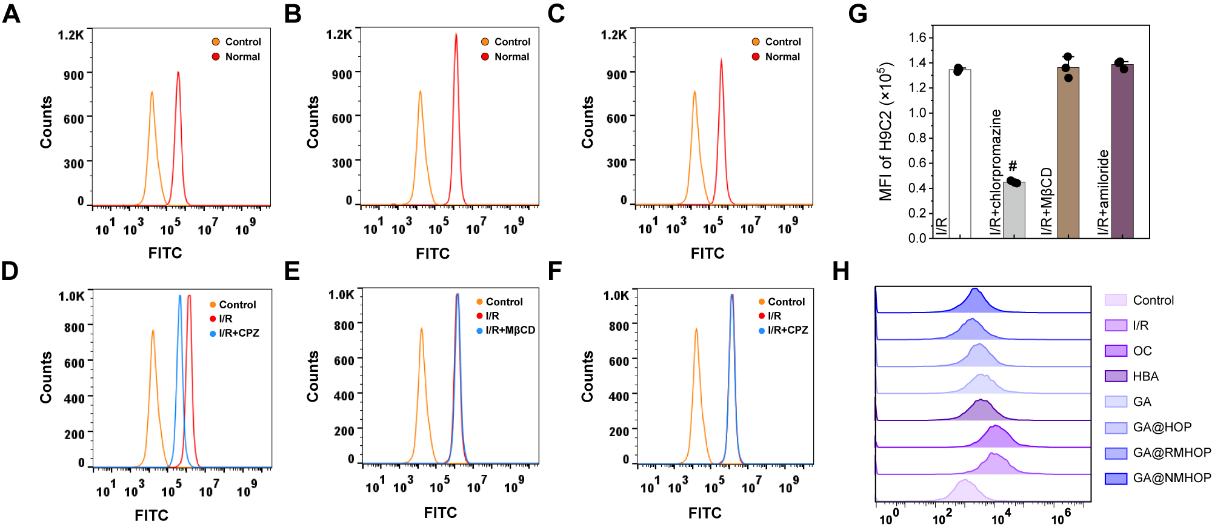** |
| --- |
| **Fig. S5.** Uptake of NDs in Cardiomyocytes. (A-C) Quantification of flow cytometry analysis of NDs uptake is shown as mean fluorescence intensity. n = 3. (D-G) Quantification of flow cytometry analysis of NDs uptake stimulated by different uptake pathway inhibitors. n = 3. (H) Quantification of DCFH-DA fluorescence by flow cytometry. ^#^*p* < 0.05 vs. I/R group. All data are shown as means ± SD. Statistical analyses were performed by one-way ANOVA followed by Tukey’s post-hoc test. |

| **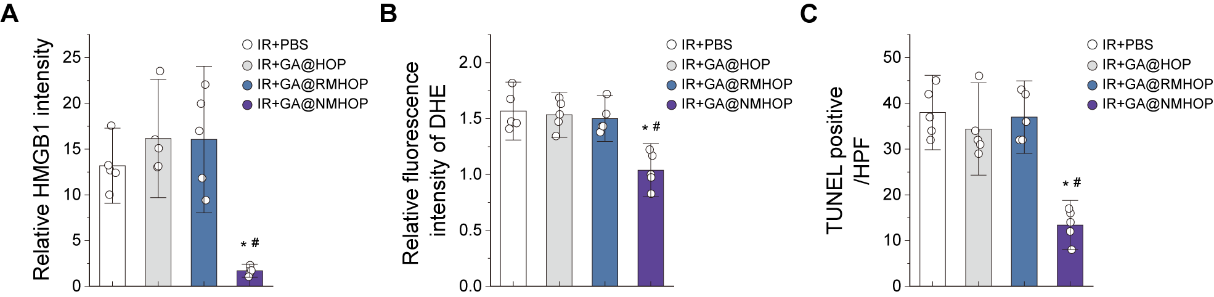** |
| --- |
| **Fig. S6.** Antioxidative and anti-inflammatory activities of NDs. (A) Quantification of HMGB1. n = 5. (B) Quantification of DHE. n = 5. (C) Statistical results of TUNEL-positive cells per field. n = 5. ^*^*p* < 0.05 vs. PBS group. All data are shown as means ± SD. Statistical analyses were performed by one-way ANOVA followed by Tukey’s post-hoc test. |
|  |
| **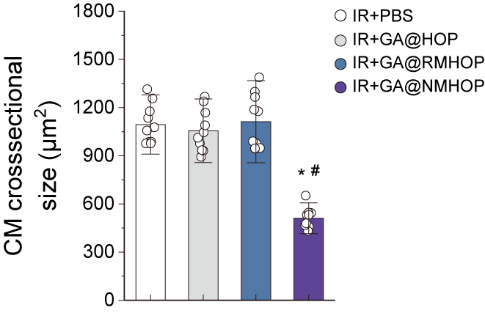** |
| **Fig. S7.** Quantitative analysis of myocyte cross-sectional area. n = 10. ^*^*p* < 0.05 vs. PBS group. All data are shown as means ± SD. Statistical analyses were performed by one-way ANOVA followed by Tukey’s post-hoc test. |

**Table S2. Primers used for qPCR (RNA)**

| Gene | Forward (5’ to 3’) | Reverse (5’ to 3’) |
| --- | --- | --- |
| IL-1β | GCAACTGTTCCTGAACTCAACT | ATCTTTTGGGGTCCGTCAACT |
| TNF-α | CTGTAGCCCACGTCGTAGC | TTGAGATCCATGCCGTTG |
| IL-6 | TAGTCCTTCCTACCCCAATTTCC | TTGGTCCTTAGCCACTCCTTC |
| iNOS | TCACCTTCGAGGGCAGCCGA | TCCGTGGCAAAGCGAGCCAG |
| HMGB1 | GGCGAGCATCCTGGCTTATC | GGCTGCTTGTCATCTGCTG |
| GAPDH | TGATGACATCAAGAAGGTGGTGAAG | TCCTTGGAGGCCATGTAGGCCAT |
